# Zoonotic diseases in China: epidemiological trends, incidence forecasting, and comparative analysis between real-world surveillance data and Global Burden of Disease 2021 estimates

**DOI:** 10.1186/s40249-025-01335-3

**Published:** 2025-07-04

**Authors:** Yun-Fei Zhang, Shi-Zhu Li, Shi-Wen Wang, Di Mu, Xi Chen, Sheng Zhou, Hai-Jian Zhou, Tian Qin, Qin Liu, Shan Lv, Yan Lu, Ji-Chun Wang, Yu Qin, Guo-Bing Yang, Yong-Jun Li, Jian-Yun Sun, Xiao-Nong Zhou, Mai-Geng Zhou, Can-Jun Zheng, Biao Kan, Shun-Xian Zhang

**Affiliations:** 1 National Key Laboratory of Intelligent Tracking and Forecasting for Infectious Diseases, National Institute for Communicable Disease Control and Prevention, Chinese Center for Disease Control and Prevention, Beijing, 102206 China; 2 National Key Laboratory of Intelligent Tracking and Forecasting for Infectious Diseases, NHC Key Laboratory of Parasite and Vector Biology, WHO Collaborating Centre for Tropical Diseases, National Center for International Research On Tropical Diseases, National Institute of Parasitic, Diseases of Chinese Center for Disease Control and Prevention, Shanghai, 200025 China; 3 National Key Laboratory of Intelligent Tracking and Forecasting for Infectious Diseases, National Institute for Viral Disease Control and Prevention, Chinese Center for Disease Control and Prevention, Beijing, 102206 China; 4 National Key Laboratory of Intelligent Tracking and Forecasting for Infectious Diseases, Chinese Center for Disease Control and Prevention, Beijing, 102206 China; 5 Gansu Provincial Center for Disease Control and Prevention, Gansu Provincial Academy of Preventive Medicine, Lanzhou, 730000 Gansu China; 6 National Center for Chronic and Noncommunicable Disease Control and Prevention, Chinese Center for Disease Control and Prevention, Beijing, 100050 China; 7 Longhua Hospital, Shanghai University of Traditional Chinese Medicine, Shanghai, 200032 China

**Keywords:** Zoonotic diseases, Incidence rate, Epidemiological trend, One health, China

## Abstract

**Background:**

Zoonotic diseases remain a significant public health challenge in China. This study examines the temporal trends, disease burden, and demographic patterns of major zoonoses from 2010 to 2023.

**Methods:**

This study analyzed data from China’s National Notifiable Infectious Disease Reporting System (NNIDRS, 2010–2023) on nine major zoonoses, including echinococcosis, brucellosis, leptospirosis, anthrax, leishmaniasis, encephalitis (Japanese encephalitis), hemorrhagic fever, rabies, and schistosomiasis. Joinpoint regression was applied to assess annual trends in incidence rates, while autoregressive integrated moving average (ARIMA) and exponential smoothing models were used to forecast incidence trends from 2024 to 2035. To assess the performance of the Global Burden of Disease (GBD) 2021 model in China, disease-specific multipliers—defined as the ratio of GBD estimates to national surveillance data—along with their corresponding 95% confidence intervals (*CI*s) were calculated to quantify discrepancies and evaluate the consistency between modeled estimates and empirical observations.

**Results:**

From 2010 to 2023, the incidence rates of leptospirosis [average annual percent change (AAPC) = − 5.527%, 95% *CI*: − 11.054, − 0.485], encephalitis (AAPC = − 16.934%, 95% *CI*: − 23.690, − 11.245), hemorrhagic fever (AAPC = − 5.384%, 95% *CI*: − 7.754, − 2.924), rabies (AAPC = − 20.428%, 95% *CI*: − 21.076, − 19.841), and schistosomiasis (AAPC = − 28.378%, 95% *CI*: − 40.688, − 15.656) showed a declining trend in China. In contrast, brucellosis exhibited a modest but statistically significant increase (AAPC = 0.151%, 95% *CI*: 0.031, 0.272). For most diseases, incidence rates were consistently higher in males than females. Children aged 0–5 years accounted for a substantial proportion of encephalitis and leishmaniasis cases, while adults aged 14–65 years represented the primary affected group across the majority of diseases. Occupationally, farmers and herders were the most affected populations. Compared to national surveillance data, the GBD 2021 model substantially overestimated the burden of zoonotic diseases in China, particularly for echinococcosis (by 3.611–7.409 times) and leishmaniasis (by 3.054–10.500 times).

**Conclusion:**

The study revealed significant decline in several major zoonoses in China, while brucellosis showed a continued upward trend. These findings highlight the urgent need for a One Health-based prevention and control system to interrupt cross-species transmission and reduce long-term public health risks.

**Supplementary Information:**

The online version contains supplementary material available at 10.1186/s40249-025-01335-3.

## Background

Zoonoses are infectious diseases that can be naturally transmitted between vertebrate animals and humans. They are caused by a broad spectrum of pathogens—including viruses, bacteria, parasites, and certain fungi—and are characterized by high biological diversity and complex ecological transmission dynamics [1–3]. Key epidemiological features of zoonotic diseases include a wide host range, strong cross-species transmission potential, and diverse routes of transmission, such as direct contact, inhalation of droplets, vector-mediated exposure, and foodborne ingestion [4, 5]. Common zoonotic pathogens encompass a wide range of organisms, including viruses (e.g., rabies virus, Ebola virus, monkeypox virus, and members of the coronavirus family), bacteria (e.g., *Brucella* spp., *Mycobacterium tuberculosis*, *Bacillus anthracis*, *Leptospira* spp., and *Yersinia pestis*), parasites (e.g., *Sparganum*, *Toxoplasma gondii*, *Echinococcus* spp., and *Schistosoma* spp.), and fungi (e.g., dermatophytes) [1, 3, 6].

Against the backdrop of ongoing human disruption to ecosystems, numerous pathogens harbored in animal hosts have acquired the capacity to breach species barriers, adapt to new hosts, and cause human infections [7]. This cross-species transmission mechanism has significantly intensified the emergence and re-emergence of infectious diseases. More than 60% of emerging infectious diseases worldwide originate from zoonotic sources. High-impact viral diseases—such as severe acute respiratory syndrome (SARS), Middle East respiratory syndrome, Ebola virus disease, Mpox, and coronavirus disease 2019 (COVID-19)—have repeatedly triggered major regional outbreaks and global public health emergencies [8, 9]. These outbreaks underscore the profound uncertainties surrounding zoonotic pathogens—including their rapid evolutionary dynamics, host adaptability, and diverse transmission routes [1, 2].

The emergence and transmission of zoonotic diseases reflect the deep coupling and adaptive complexity between ecological and social systems under the pressures of global change. Their transmission dynamics are driven by multi-faceted interactions between environmental factors and human activities, continuously evolving through the interconnected networks of pathogens, hosts, vectors, and human populations [1, 2]. At the ecosystem level, habitat destruction, degradation, and spatial fragmentation have markedly altered the geographic range and behavioral patterns of wildlife, increased the frequency of human-wildlife contact and amplified the risk of pathogen niche spillover. Simultaneously, land-use changes—such as agricultural expansion, urbanization, and deforestation—are shrinking natural ecological buffers and weakening the regulatory functions of ecosystems, thereby facilitating cross-species transmission of pathogens [1, 2]. Climate change, as a key driving force, alters temperature, precipitation, and humidity patterns, thereby reshaping the life cycle dynamics of pathogens, the reproduction and survival rates of vector species, and the ecological behavior and geographic distribution of host animals. These environmental perturbations are facilitating the expansion of traditionally tropical zoonoses—such as dengue, plague, and Rift Valley fever—into temperate and higher-latitude regions, triggering emerging and re-emerging outbreaks [10, 11].

In the context of globalization, profound shifts in human social activity have markedly reshaped the transmission patterns and control challenges of zoonotic diseases. The increasing interconnectedness of global trade and transportation networks has facilitated the rapid movement of people, animals, pathogens, and vectors across national and regional boundaries, eroding traditional geographic barriers to disease spread and accelerating the emergence of transboundary epidemics [12, 13]. Concurrently, rapid population mobility, urban migration, and accelerating urbanization have led to the concentration of dense human populations—particularly in marginalized communities and peri-urban settlements with inadequate public health infrastructure—amplifying exposure risks among vulnerable groups. Spatial disparities in health resource allocation have further widened the capacity gap between the global North and South, as well as within countries, exacerbating structural inequalities in disease prevention and control [13–15].

Zoonotic diseases pose a significant and growing public health threat globally [1, 2]. In China, although substantial progress has been made in strengthening infectious disease control systems, emerging socio-ecological dynamics continue to complicate the epidemiological landscape [16]. Rapid urban–rural disparities, a structural shift in livestock production from small-scale to high-density industrialized systems, increased human mobility, and the expansion of transportation networks have collectively driven the co-circulation, cross-regional spread, and seasonal resurgence of multiple zoonoses [17]. These factors not only heighten the risk of cross-species transmission but also intensify geographic, ecological, and social health vulnerabilities, thereby undermining early warning capabilities, equitable resource allocation, and the implementation of targeted interventions.

The Global Burden of Disease Study (GBD) 2021 represents the most comprehensive and methodologically rigorous initiative to quantify health loss at the global level. Encompassing 371 diseases and injuries and 88 risk factors across 204 countries and territories [18, 19]. At the national level, burden estimates for major infectious diseases of public health importance are derived from multiple data sources, including vital registration systems, population censuses, household surveys, disease-specific registries, health service utilization data, and so on [18, 19]. However, several important infectious diseases—such as brucellosis, hemorrhagic fever, schistosomiasis, leptospirosis, and anthrax—remain absent from the GBD 2021 estimation framework, despite their high relevance in endemic regions. Consequently, there is a lack of high-resolution, multi-disease burden data for zoonoses, which hinders a systematic understanding of their spatiotemporal transmission patterns and health impacts among different populations. This data gap constrains the accurate identification of epidemiological dynamics and impedes evidence-based decision-making for resource allocation and intervention strategies. To address this limitation, national surveillance data from China were utilized through the National Notifiable Infectious Disease Reporting System (NNIDRS), a real-time, internet-based platform, to conduct a comprehensive and longitudinal assessment of the burden of zoonotic diseases in China. By characterizing spatial heterogeneity, temporal trends, and key driving factors, this study aims to inform the optimization of cross-sectoral control strategies and strengthen the resilience of the public health system.

## Methods

### Surveillance data

Data in the study was drawn from the NNIDRS. NNIDRS was established in 2003 and is an internet-based, long-term surveillance system that covers more than 85% of all health facilities in China, and its details were reported in the previous studies (Additional file 1: Fig. S1) [16, 20, 21]. The NNIDRS captures data on all notifiable infectious diseases in Chinese mainland (not included Hong Kong, Macao, and Taiwan), covering both sporadic cases and outbreaks, with detailed individual case information. The system mandates that cases of cholera, plague, anthrax, and SARS be reported by the attending physician within 2 h at the first encounter, while all other notifiable diseases must be reported within 24 h. Upon diagnosing a probable, clinical, or laboratory-confirmed case, physicians are required to complete a standardized reporting form and submit it directly to the NNIDRS [16]. Case report information is initially reviewed and submitted online by infectious disease management personnel at medical institutions. Regular internal audits are conducted to ensure the quality of epidemic reporting. Public health professionals at local Centers for Disease Control and Prevention (CDC) further verify and confirm the quality of the web-based reports through their epidemic management divisions, thereby finalizing the reported cases. Subsequently, local epidemiologists conduct field investigations using a standardized form, which captures essential demographic characteristics, case classification, dates of symptom onset and diagnosis, and clinical outcomes, including death if applicable [16, 20, 21].

Case data for nine priority zoonotic diseases—echinococcosis, brucellosis, leptospirosis, anthrax, leishmaniasis, encephalitis (Japanese encephalitis), hemorrhagic fever, rabies, and schistosomiasis—spanning from 1 January 2010 to 31 December 31 2023 were extracted from NNIDRS [22, 23]. Records identified as suspected cases or asymptomatic carriers were excluded, only laboratory-confirmed and clinically diagnosed cases were retained in the study. In addition, within real-time infectious disease surveillance database, public health physicians at local (county-level) CDC regularly review and correct duplicate case reports for example, cases in which the same infectious disease has been reported by multiple medical institutions [16]. Moreover, all sentinel hospitals are required to report at least 95% of diagnosed cases of notifiable infectious diseases encountered during clinical visits.

The NNIDRS collects comprehensive individual-level data for all notifiable infectious diseases reported in China. Each reported case includes detailed demographic information (e.g., age, sex, occupation, and residence), clinical and diagnostic classifications (e.g., date of onset, diagnosis, and case type), and reporting details (e.g., reporting institution, physician, and time of report). When available, laboratory test results and sample types are also documented. These data enable real-time surveillance, support outbreak investigations, and facilitate targeted public health interventions at both national and local levels [16]. In additions, population data from 2010 to 2023 were obtained from the China CDC Information System and the China Statistical Yearbook published by the National Bureau of Statistics [24]. Disease incidence at national levels was calculated by dividing the number of reported cases for each disease by the corresponding population size, and presented as per 100,000 population [16, 22, 23].

### GDB 2021 data

The GBD 2021 systematically adjusted raw epidemiological data to account for potential biases arising from heterogeneity in data sources, case definitions, and measurement methodologies. These adjustments were implemented through advanced statistical modeling frameworks, notably the Meta-Regression–Bayesian, Regularized, Trimmed platform and DisMod-MR 2.1, both of which incorporate Bayesian priors, regularization techniques, and trimming algorithms. These tools facilitate the harmonization of data across geographic regions, age groups, sexes, and time periods, thereby ensuring internal consistency and methodological coherence across all estimates. Furthermore, standardized calibration procedures were applied to minimize cross-source variability and enhance the comparability of disease burden metrics [25, 26].

Estimates of incidence rate and counts in GBD 2021 were derived from a diverse array of data sources, including national disease surveillance systems, population-based health surveys, and peer-reviewed literature. In this study, annual incidence rates, case counts, and associated 95% uncertainty intervals (UIs) for the period of 2010–2021 were extracted using the publicly available GBD Results Tool (https://vizhub.healthdata.org/gbd-results/). Four zoonotic diseases—echinococcosis, leishmaniasis, encephalitis, and rabies—were selected for analysis because they are the only ones that are consistently documented in both the GBD 2021 dataset and China’s national real-time infectious disease surveillance system.

### Statistical analysis

The disease burden of zoonotic diseases based on data from the NNIDRS was assessed using both incidence rates and case counts, stratified by year, sex, occupation, and other demographic variables. Incidence rates, expressed as per 100,000 population, reflect the relative burden of disease, whereas case counts indicate the absolute burden. Both measures were reported with corresponding 95% confidence intervals (*CI*s) [27–29]. In parallel, the burden estimates derived from the GBD 2021 database were presented as incidence rates accompanied by 95% UIs.

To quantify the discrepancies between modelled estimates and empirical data, we computed the fold difference, defined as the ratio of the GBD 2021-estimated incidence rate to the corresponding incidence rate reported in national surveillance data for each disease and year. This was calculated as follows:

$${\text{Fold difference}}\, = \,{\text{Incidence rate 2}}0{21}/{\text{surveillance data}}$$

All statistical analyses were performed using R software (version 4.4.1; R Foundation for Statistical Computing, Vienna, Austria; available at https://cran.r-project.org). Detailed descriptions of the analytical approaches—such as estimation of the annual percentage change (EAPC), Joinpoint regression analysis, and forecasting models—are available in previously published studies [18, 27–30].

#### EAPC

The temporal trends in the incidence rates of the nine infectious diseases from 2010 to 2023, and projected from 2024 to 2035, were quantified using the EAPC. The EAPC was calculated based on the following regression model [29, 31, 32]:

$${\text{ln}}\left( y \right)\, = \,\alpha \, + \,\beta .\chi$$

$${\text{EAPC}}\, = \,\left( {{\text{e}}^{\beta } {-}{1}} \right)\, \times \,{1}00\%$$

where y represents the disease burden metric, a is intercept term, β is slope coefficient, *χ* is year. An EAPC value less than 0, with the upper bound of its 95% *CIs* also below 0, indicates a declining trend. Conversely, an EAPC value greater than 0, with the lower bound of its 95% *CIs* above 0, signifies an increasing trend.

### Joinpoint regression analysis

To account for potential structural changes in incidence trends over time, the average annual percentage change (AAPC) was estimated using segmented log-linear regression models. Specifically, the AAPC represents a weighted average of the annual percentage changes (APCs) across multiple time segments, with weights proportional to the duration of each segment. A grid search method was used to calculate all possible breakpoints, selecting the one with the minimum mean squared error (MSE) as the optimal breakpoint. The number of optimal breakpoints was further determined through a Monte Carlo permutation test, allowing for a range of 0 to 5 breakpoints [28, 29].

$${\text{APC}}_{i} = ({\text{e}}^{{\beta_{i} }} - 1) \times 100{\text{\% }}$$

$${\text{AAPC}}_{i} = {\text{(e}}^{{\frac{{\sum {W_{i} \beta_{i} } }}{{\sum {W_{i} } }}}} - 1) \times 100{\text{\% }}$$

In this model, $i$ is the number of segments, $\beta_{i}$ is the regression coefficient from the log-linear model ln(*y*) = *β* × year + constant. $W_{i}$ represented by the length of each corresponding segment. When the AAPC is greater than 0 with a *P* value less than 0.05, it indicates a statistically significant upward trend. Conversely, an AAPC less than 0 with a *P* value below 0.05 denotes a statistically significant downward trend. The AAPC reflects the overall trend by weighting the APC for each segment based on the duration of the respective time spans. This analytical approach not only improves the precision of trend identification over time but also enhances the robustness of the model.

### Forecasting models

Based on incidence data from 2010 to 2023 for nine zoonotic diseases, predictive models were developed using ARIMA and exponential smoothing approaches, including Holt’s linear trend, Brown’s linear trend, and the damped trend model [30, 33]. These models were applied to estimate disease incidence for both the historical period (2010–2023) and the future period (2024–2035).

Model selection was guided by a comprehensive evaluation of performance metrics, including the adjusted coefficient of determination (adjusted *R*^2^, with higher values indicating better model fit), the normalized Bayesian information criterion (NBIC, where lower values indicate better model parsimony), the root mean square error (RMSE, with lower values indicating higher accuracy), and the mean absolute percentage error (MAPE, also preferred to be as low as possible). In addition, statistical significance of model parameters (*P* < 0.05) was required to ensure the validity of the model. For ARIMA models, at least one of the autoregressive (AR) or moving average (MA) parameters—or both—had to be statistically significant (*P* < 0.05) [30]. Among exponential models, Holt’s linear trend includes two parameters: *α* (level) and *β* (trend); Brown’s linear trend includes only a single parameter *α*; while the damped trend model incorporates three parameters: *α*, *β*, and *φ* (damping factor). Among models that met all diagnostic criteria, the most parsimonious model—with the fewest statistically significant parameters—was considered optimal (Additional file 1) [33–35].

When optimizing and selecting models, particular attention must be paid to their predictive performance—that is, the model’s ability to accurately forecast real-world data. In this study, predictive accuracy was assessed by calculating the mean relative error (MRE) between the reported incidence rates and the model-predicted values for the period 2010–2023. A low MRE indicates higher predictive accuracy and, consequently, great practical value and applicability of the model [36].

$${\text{MER}} = \frac{1}{N}\sum\limits_{t = 2010}^{2023} {\frac{{{|}\chi_{{2}} {-}\chi_{{1}} {|}}}{{\chi_{{1}} }}}$$

In the analysis, *N* = 14, $\chi_{1}$ is predicted value, $\chi_{2}$ is reported value. MRE does not have a universally fixed threshold, as its acceptable range depends on the research context, data characteristics, and domain-specific standards. However, in the field of disease modeling and public health, an MRE below 10% is generally considered excellent, indicating highly accurate predictions. An MRE between 10 and 20% is regarded as good, reflecting a reasonable level of predictive accuracy. Values between 20 and 50% are usually considered acceptable, especially in the context of low-incidence or highly variable diseases. In contrast, an MRE above 50% suggests poor model performance and indicates the need for further optimization or model refinement [36].

Once the optimal models were identified, they were used to project the incidence rates of the nine zoonotic diseases for the period of 2024–2035. To evaluate the temporal trends in disease burden over this future period, AAPC and EAPC were calculated (Additional file 1).

## Results

### Epidemiological trends

#### Echinococcosis

**Fig. 1:**
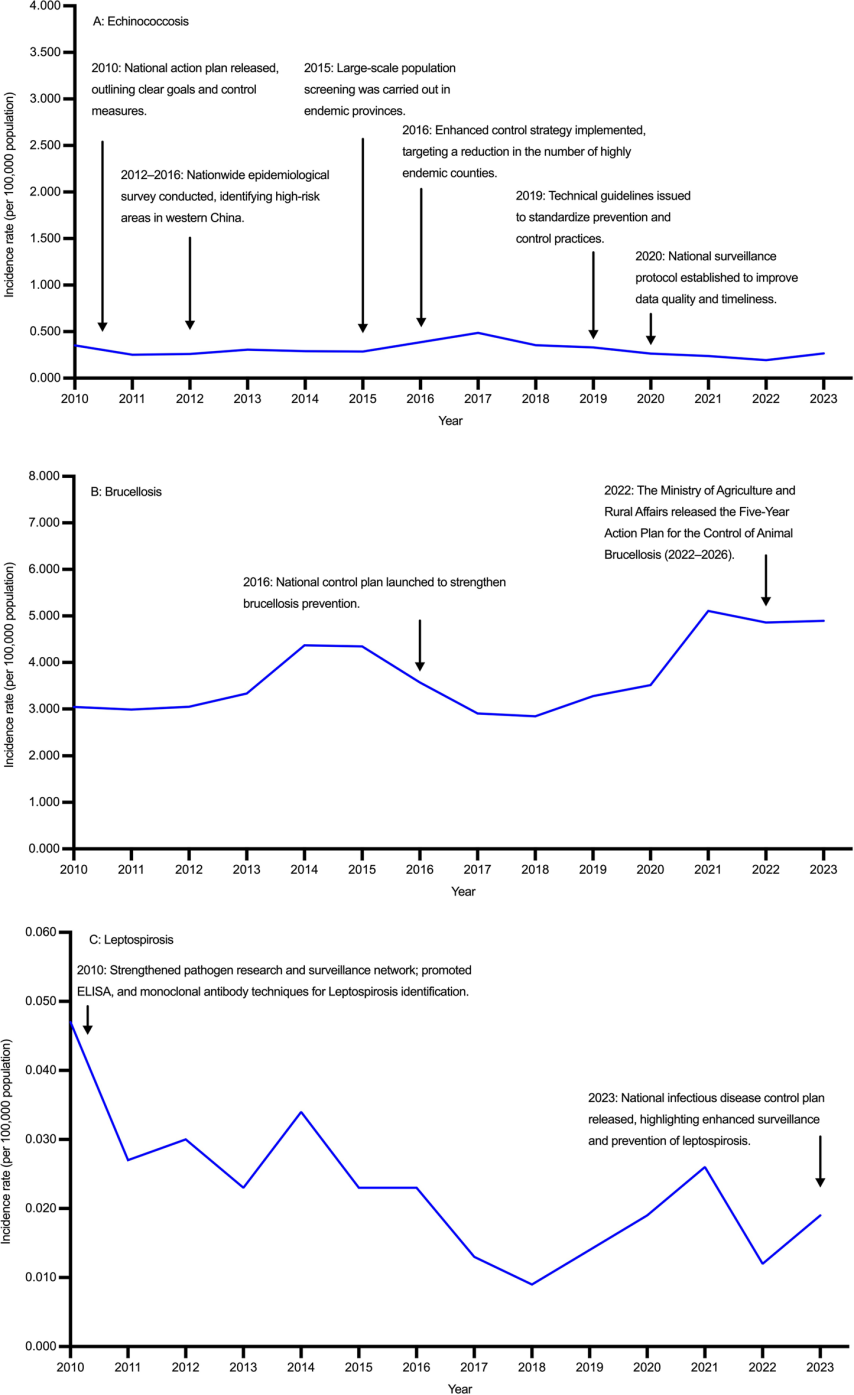

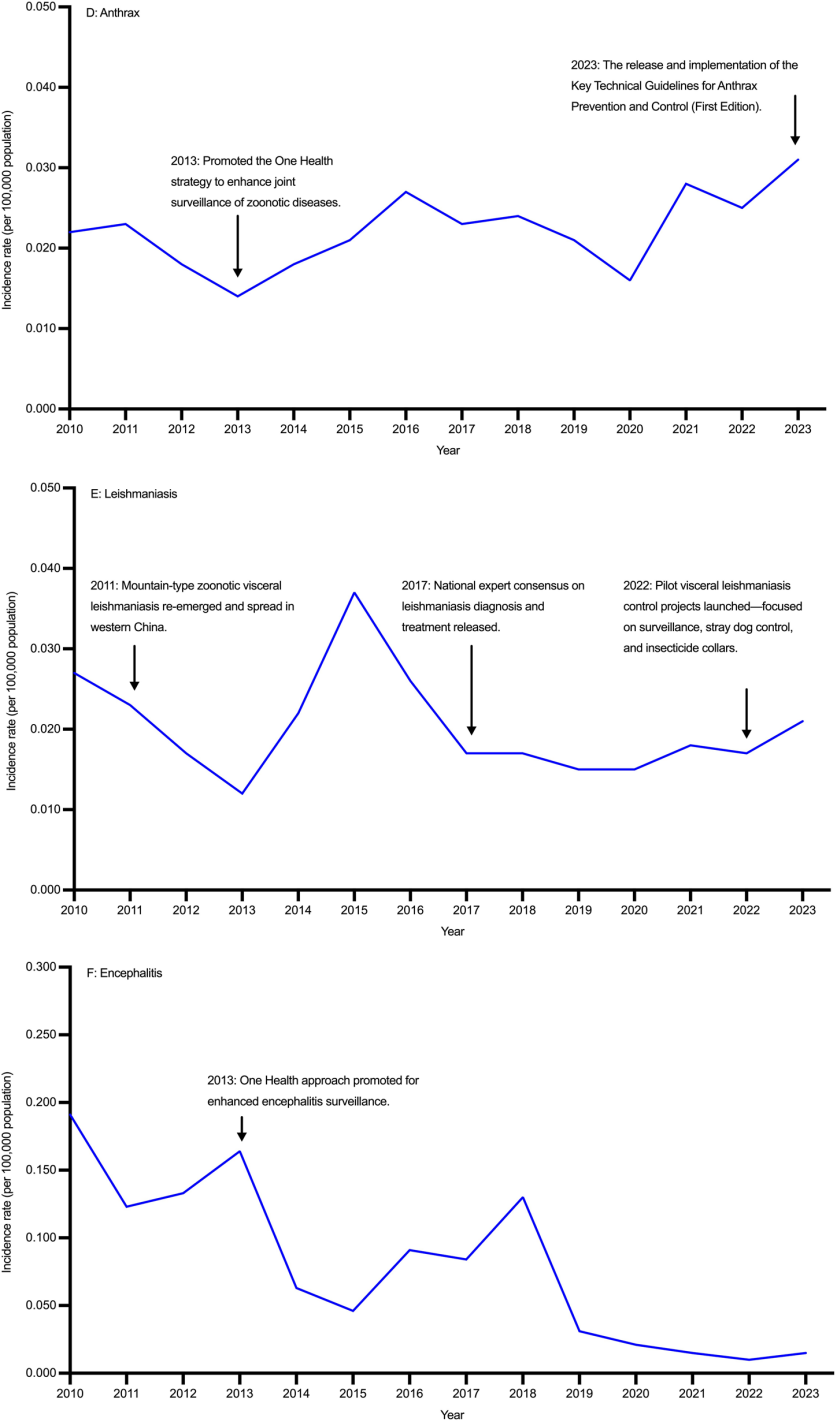

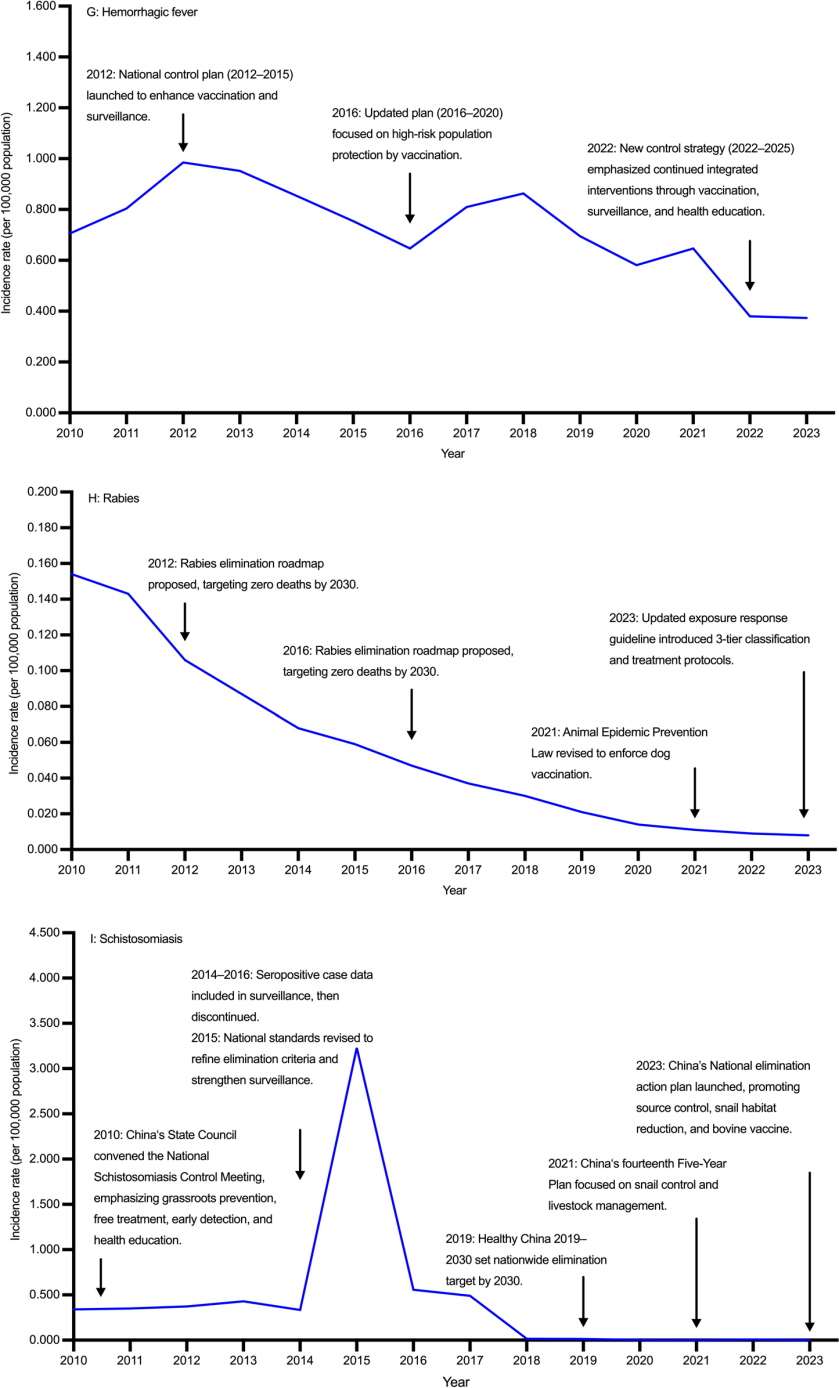
Temporal trends of nine zoonotic diseases in China, 2010–2023 (**A** Echinococcosis, **B** Brucellosis, **C** Leptospirosis, **D** Anthrax, **E** Leishmaniasis, **F** Encephalitis, **G** Hemorrhagic fever, **H** Rabies, **I** Schistosomiasis)

From 2010 to 2023, the incidence rate of echinococcosis in China showed a fluctuating pattern without significant overall decline (AAPC = –1.848%, 95% *CI*: − 6.102, 2.474), increasing from 0.352 per 100,000 population in 2010 to a peak of 0.486 per 100,000 population in 2017, and then decreasing to 0.265 per 100,000 population in 2023. Females consistently had higher incidence rates than males. Although AAPCs for both sexes were not statistically significant over the entire period, a marked decline was observed during 2018–2023. Reported cases declined from 4702 in 2010 to 3734 in 2023. Females accounted for more cases than males throughout the study period (Tables 1, 2, Fig. 1A; Additional file 1: Table S1, Table S2, Fig.S2A).

**Table 1:** Incidence rates of nine zoonotic diseases in both gender in China, 2010–2023

Year	Echinococcosis, incidence rate (per 100,000 population) (95% *CI*)	Brucellosis, incidence rate (per 100,000 population) (95% *CI*)	Leptospirosis, incidence rate (per 100,000 population) (95% *CI*)	Anthrax, incidence rate (per 100,000 population) (95% *CI*)	Leishmaniasis, incidence rate (per 100,000 population) (95% *CI*)	Encephalitis, incidence rate (per 100,000 population) (95% *CI*)	Hemorrhagic fever, incidence rate (per 100,000 population) (95% *CI*)	Rabies, incidence rate (per 100,000 population) (95% *CI*)	Schistosomiasis, incidence rate (per 100,000 population) (95% *CI*)
2010	0.352 (0.342, 0.362)	3.044 (3.014, 3.073)	0.047 (0.047, 0.055)	0.022 (0.019, 0.024)	0.027 (0.024, 0.029)	0.191 (0.184, 0.199)	0.706 (0.692, 0.720)	0.191 (0.184, 0.199)	0.339 (0.329, 0.349)
2011	0.251 (0.242, 0.259)	2.990 (2.961, 3.019)	0.027 (0.027, 0.033)	0.023 (0.020, 0.026)	0.023 (0.020, 0.025)	0.123 (0.117, 0.129)	0.804 (0.789, 0.820)	0.123 (0.117, 0.129)	0.349 (0.339, 0.359)
2012	0.259 (0.251, 0.268)	3.050 (3.021, 3.080)	0.030 (0.030, 0.036)	0.018 (0.015, 0.020)	0.017 (0.015, 0.019)	0.133 (0.127, 0.139)	0.985 (0.968, 1.001)	0.133 (0.127, 0.139)	0.372 (0.361, 0.382)
2013	0.307 (0.298, 0.317)	3.332 (3.301, 3.363)	0.023 (0.023, 0.029)	0.014 (0.012, 0.016)	0.012 (0.010, 0.014)	0.164 (0.157, 0.171)	0.952 (0.936, 0.968)	0.164 (0.157, 0.171)	0.429 (0.418, 0.440)
2014	0.291 (0.282, 0.300)	4.371 (4.336, 4.406)	0.034 (0.034, 0.040)	0.018 (0.016, 0.020)	0.022 (0.019, 0.024)	0.063 (0.059, 0.067)	0.854 (0.839, 0.870)	0.063 (0.059, 0.067)	0.333 (0.324, 0.343)
2015	0.286 (0.277, 0.295)	4.344 (4.309, 4.379)	0.023 (0.023, 0.029)	0.021 (0.019, 0.024)	0.037 (0.034, 0.041)	0.046 (0.042, 0.050)	0.754 (0.740, 0.769)	0.046 (0.042, 0.050)	3.229 (3.199, 3.259)
2016	0.386 (0.376, 0.396)	3.569 (3.537, 3.600)	0.023 (0.023, 0.028)	0.027 (0.024, 0.030)	0.026 (0.024, 0.029)	0.091 (0.086, 0.096)	0.647 (0.634, 0.661)	0.091 (0.086, 0.096)	0.556 (0.543, 0.568)
2017	0.486 (0.474, 0.497)	2.906 (2.877, 2.934)	0.013 (0.013, 0.018)	0.023 (0.021, 0.026)	0.017 (0.015, 0.019)	0.084 (0.079, 0.088)	0.810 (0.795, 0.825)	0.084 (0.079, 0.088)	0.489 (0.477, 0.500)
2018	0.354 (0.344, 0.364)	2.842 (2.814, 2.870)	0.009 (0.009, 0.013)	0.024 (0.021, 0.027)	0.017 (0.014, 0.019)	0.130 (0.124, 0.136)	0.863 (0.848, 0.879)	0.130 (0.124, 0.136)	0.016 (0.014, 0.018)
2019	0.331 (0.322, 0.341)	3.277 (3.247, 3.307)	0.014 (0.014, 0.018)	0.021 (0.019, 0.024)	0.015 (0.013, 0.017)	0.031 (0.028, 0.033)	0.695 (0.681, 0.708)	0.031 (0.028, 0.033)	0.014 (0.012, 0.016)
2020	0.263 (0.254, 0.271)	3.515 (3.484, 3.546)	0.019 (0.019, 0.024)	0.016 (0.014, 0.018)	0.015 (0.013, 0.018)	0.021 (0.018, 0.023)	0.581 (0.569, 0.594)	0.021 (0.018, 0.023)	0.004 (0.003, 0.005)
2021	0.237 (0.229, 0.245)	5.110 (5.072, 5.147)	0.026 (0.026, 0.031)	0.028 (0.025, 0.030)	0.018 (0.015, 0.020)	0.015 (0.013, 0.017)	0.647 (0.634, 0.661)	0.015 (0.013, 0.017)	0.001 (0.001, 0.002)
2022	0.194 (0.187, 0.202)	4.861 (4.824, 4.897)	0.012 (0.012, 0.015)	0.025 (0.022, 0.027)	0.017 (0.015, 0.019)	0.010 (0.009, 0.012)	0.380 (0.370, 0.390)	0.010 (0.009, 0.012)	0.007 (0.005, 0.008)
2023	0.265 (0.256, 0.273)	4.894 (4.858, 4.931)	0.019 (0.019, 0.024)	0.031 (0.028, 0.034)	0.021 (0.019, 0.024)	0.015 (0.013, 0.017)	0.373 (0.363, 0.383)	0.015 (0.013, 0.017)	0.002 (0.001, 0.002)

Data are derived from the National Notifiable Infectious Disease Reporting System of China. “Encephalitis” typically refers to Japanese encephalitis, also known as epidemic Japanese encephalitis, which is an acute zoonotic infectious disease caused by the Japanese encephalitis virus. From 2010 to 2023, nearly all anthrax cases reported through China’s national surveillance system were cutaneous anthrax, with only five cases of pulmonary anthrax documented during this period

*APC* annual percent change, *AAPC* average annual percent change, *CI* confidence interval

**Table 2:** Temporal trends of echinococcosis, brucellosis, and leptospirosis in China, 2010–2023

Gender	Index	Echinococcosis		Brucellosis		Leptospirosis	
		Period	Value (%) (95%* CI*)	Period	Value (%) (95% *CI*)	Period	Value (%) (95% *CI*)
Both gender	AAPC	2010–2023	− 1.848 (− 6.102, 2.474)	2010–2023	0.151 (0.031, 0.272)	2010–2023	− 5.527 (− 11.054, − 0.485)
	APC	2010–2017	5.470 (− 0.046, 11.289)	2010–2019	0.361 (− 3.713, 4.607)	2010–2018	− 12.345 (− 38.854, 20.134)
	APC	2018–2023	− 9.746 (− 16.952, − 1.915)	2020–2023	11.908 (1.107, 23.865)	2018–2023	6.503 (− 13.253, 59.366)
Male	AAPC	2010–2023	− 2.093 (− 7.547, 3.527)	2010–2023	0.186 (0.056, 0.316)	2010–2023	− 3.836 (− 9.854, 1.838)
	APC	2010–2017	4.918 (− 1.388, 45.578)	2010–2019	− 0.060 (− 4.155, 4.210)	2010–2018	− 10.860 (− 39.223, 34.315)
	APC	2018–2023	− 9.683 (− 38.806, − 2.280)	2020–2023	11.551 (0.685, 23.589)	2018–2023	8.573 (− 25.222, 64.444)
Female	AAPC	2010–2023	− 1.616 (− 5.710, 2.518)	2010–2023	0.106 (0.057, 0.156)	2010–2023	− 9.786 (− 18.563, − 0.827)
	APC	2010–2017	5.642 (− 0.057, 29.948)	2010–2019	1.575 (− 2.487, 5.806)	2010–2018	− 16.584 (− 51.895, 64.496)
	APC	2018–2023	− 9.600 (− 28.616, − 3.034)	2020–2023	12.703 (1.982, 24.552)	2018–2023	2.261 (− 46.345, 83.941)

*APC* annual percent change, *AAPC* average annual percent change, *CI* confidence interval

#### Brucellosis

From 2010 to 2023, the incidence rate of brucellosis in China showed a generally increasing trend (AAPC = 0.151%, 95% *CI*: 0.031, 0.272). Joinpoint regression identified a non-significant change during 2010–2019, followed by a significant increase during 2020–2023. Incidence rates were consistently higher in males than in females. No significant changes were detected in either sex during 2010–2019, while male and female showed marked increases from 2020 to 2023. Reported cases fluctuated during the study period, with a notable rise in 2014–2015, decline by 2018, and resurgence to 72,036 cases in 2021. Males consistently accounted for over 70% of cases annually (Tables 1, 2; Fig. 1B; Additional file 1: Table S1, Table S2, Fig.S2B).

#### Leptospirosis

From 2010 to 2023, the incidence rate of leptospirosis in China showed an overall decline (AAPC = − 5.527%, 95% *CI*: − 11.054, − 0.485), decreasing from 0.047 per 100,000 population in 2010 to 0.009 per 100,000 population in 2018, with a slight rebound to 0.019 per 100,000 population in 2023. Incidence rates remained consistently higher in males than in females. No significant reduction was observed among males (AAPC = − 3.836%, 95% *CI*: − 9.854, 1.838), whereas females experienced a significant decline (AAPC = –9.786%, 95% *CI*: − 18.563, − 0.827). Reported cases decreased from 679 in 2010 to 156 in 2018, followed by a modest rise to 304 in 2023, with male cases consistently outnumbering females (Tables 1, 2; Fig. 1C; Additional file 1: Table S1, Table S2, Fig.S2C).

#### Anthrax

From 2010 to 2023, the incidence rate of anthrax in China remained stable without significant fluctuations (AAPC = 0.501%, 95% *CI*: − 3.566, 5.008). Incidence rates were consistently higher in males than in females. Both sexes showed slow and non-significant variations over time, with no clear upward or downward trends. Reported cases increased from 292 in 2010 to 435 in 2023 (Tables 1, 3; Fig. 1D; Additional file 1: Table S1, Table S2, Fig.S2D).

**Table 3:** Temporal trends of anthrax, leishmaniasis, and encephalitis in China, 2010–2023

Gender	Index	Anthrax		Leishmaniasis		Encephalitis	
		Period	Value (%) (95% *CI*)	Period	Value (%) (95% *CI*)	Period	Value (%) (95% *CI*)
Both gender	AAPC	2010–2023	0.501 (− 3.566, 5.008)	2010–2023	− 4.453 (− 10.103, 2.827)	2010–2023	− 16.934 (− 23.690, − 11.245)
	APC	2010–2013	− 13.301 (− 34.394, 8.207)	2010–2012	− 28.487 (− 51.543, 16.578)	2010–2018	− 8.689 (− 38.160, 34.789)
	APC	2013–2016	14.471 (− 9.861, 32.421)	2012–2015	18.500 (− 26.276, 51.389)	2018–2021	− 44.814 (− 57.662, 21.808)
	APC	2016–2023	1.259 (− 23.829, 22.550)	2015–2023	− 5.243 (− 39.313, 35.005)	2021–2023	5.051 (− 40.268, 65.555)
Male	AAPC	2010–2023	4.540 (− 2.758, 12.100)	2010–2023	− 3.188 (− 8.861, 3.742)	2010–2023	− 17.403 (− 23.925, − 11.856)
	APC	2010–2017	4.120 (− 29.396, 71.802)	2010–2012	− 27.293 (− 49.912, 14.608)	2010–2018	− 9.497 (− 40.015, 40.896)
	APC	2017–2020	− 9.059 (− 30.210, 33.674)	2012–2015	14.273 (− 22.847, 45.655)	2018–2021	− 42.745 (− 56.420, 23.420)
	APC	2020–2023	21.309 (− 20.758, 93.954)	2015–2023	− 2.273 (− 37.800, 37.874)	2021–2023	− 0.711 (− 41.454, 57.258)
Female	AAPC	2010–2023	1.059 (− 1.468, 4.568)	2010–2023	− 26.634 (− 37.637, − 14.763)	2010–2023	− 16.314 (− 23.979, − 9.973)
	APC	2010–2013	− 11.531 (− 27.842, 2.241)	2010–2012	− 6.894 (− 12.868, 1.072)	2010–2018	− 7.687 (− 41.084, 39.564)
	APC	2013–2023	5.175 (1.619, 20.315)	2012–2015	− 30.791 (− 55.626, 20.685)	2018–2021	− 47.569 (− 61.256, 26.978)
	APC	–	–	2015–2023	26.122 (− 33.629, 65.733)	2021–2023	13.971 (− 41.395, 91.361)

Data are derived from the National Notifiable Infectious Disease Reporting System of China. The symbol ‘–’ indicates data not available or not estimable

*APC* annual percent change, *AAPC* average annual percent change, *CI* confidence interval

#### Leishmaniasis

From 2010 to 2023, the incidence rate of leishmaniasis in China showed an overall declining trend (AAPC = –4.453%, 95% *CI*: − 10.103, 2.827), with a significant decrease during 2010–2012, a short-term rise in 2012–2015, and a non-significant decline thereafter. Incidence rates were consistently higher in males than those in females. No significant change was observed in males, whereas females experienced a significant decrease. Reported cases declined from 354 in 2010 to 164 in 2013, with a modest rebound to 299 in 2023, reflecting a fluctuating upward trend in recent years. Male cases consistently outnumbered female cases (Tables 1, 3; Fig. 1E; Additional file 1: Table S1, Table S2, Fig.S2E).

#### Encephalitis

From 2010 to 2023, the incidence rate of encephalitis in China declined markedly from 0.191 to 0.015 per 100,000 population (AAPC = − 16.934%, 95% *CI*: − 23.648, − 11.245). A modest downward fluctuation was observed during 2010–2018, followed by a sharper but non-significant decline in 2018–2021, and a slight upward shift thereafter. Incidence rates were consistently higher in males than in females, with both sexes showing a downward trend over time. Reported cases decreased from 2552 in 2010 to 205 in 2023, with male cases consistently exceeding female cases (Tables 1, 3; Fig. 1F; Additional file 1: Table S1, Table S2, Fig.S2F).

#### Hemorrhagic fever

From 2010 to 2023, the incidence rate of hemorrhagic fever in China showed a significant overall decline (AAPC = − 5.384%, 95% *CI*: − 7.540, − 2.924), increasing from 0.706 per 100,000 population in 2010 to 0.985 per 100,000 population in 2012, then falling to 0.373 per 100,000 population in 2023. Joinpoint regression identified three phases: an initial increase (2010–2012), a moderate decline (2012–2021), and a sharp decrease (2021–2023). Incidence rates were consistently higher in males than in females, and the greatest decline in both sexes occurred during 2021–2023. Reported cases rose from 9423 in 2010 to 13,267 in 2012, before decreasing to 5257 in 2023. Male cases predominated throughout the study period (Tables 1, 4; Fig. 1G; Additional file 1: Table S1, Table S2, Fig.S2G).

**Table 4:** Temporal trends of hemorrhagic fever, rabies, and schistosomiasis in China, 2010–2023

Gender	Index	Hemorrhagic fever		Rabies		Schistosomiasis	
		Period	Value (%) (95% *CI*)	Period	Value (%) (95% *CI*)	Period	Value (%) (95% *CI*)
Both gender	AAPC	2010–2023	− 5.384 (− 7.754, − 2.924)	2010–2023	− 20.428 (− 21.076, − 19.841)	2010–2023	− 28.378 (− 40.688, − 15.656)
	APC	2010–2012	16.818 (− 0.415, 34.900)	2010–2018	− 18.984 (− 19.738, − 17.764)	2010–2015	46.177 (− 17.288, 243.564)
	APC	2012–2021	− 4.941 (− 8.195, − 1.351)	2018–2021	− 29.367 (− 31.632, − 25.989)	2015–2021	− 68.371 (− 88.048, 20.666)
	APC	2021–2023	− 24.959 (− 35.251, − 11.435)	2021–2023	− 11.461 (− 17.115, − 6.282)	2021–2023	39.753 (− 63.844, 297.596)
Male	AAPC	2010–2023	− 5.773 (− 8.153, − 3.334)	2010–2023	− 20.927 (− 22.016, − 20.107)	2010–2023	− 36.885 (− 55.840, − 10.442)
	APC	2010–2012	15.392 (− 1.424, 33.363)	2010–2018	− 18.815 (− 21.117, − 16.606)	2010–2015	35.157 (− 28.643, 1183.926)
	APC	2012–2021	− 5.191 (− 8.461, − 1.204)	2018–2021	− 29.683 (− 32.835, − 17.677)	2015–2023	− 60.786 (− 92.703, − 46.265)
	APC	2021–2023	− 25.157 (-35.460, − 11.687)	2021–2023	− 15.142 (− 25.257, − 8.352)	–	–
Female	AAPC	2010–2023	− 4.226 (− 6.844, − 1.507)	2010–2023	− 19.222 (− 20.872, − 17.847)	2010–2023	− 26.634 (− 37.637, − 14.763)
	APC	2010–2012	21.299 (2.097, 42.314)	2010–2017	− 18.657 (− 20.499, − 12.134)	2010–2015	38.861 (− 9.602, 180.307)
	APC	2012–2021	− 4.216 (− 7.528, − 0.543)	2017–2021	− 26.962 (− 33.766, − 22.618)	2015–2021	− 68.611 (− 86.366, − 55.777)
	APC	2021–2023	− 24.415 (− 35.619, − 10.078)	2021–2023	− 3.571 (− 18.786, 8.776)	2021–2023	90.068 (− 48.593, 402.337)

The symbol ‘–’ indicates data not available or not estimable

*APC* annual percent change, *AAPC* average annual percent change, *CI* confidence interval

#### Rabies

From 2010 to 2023, the incidence rate of rabies in China declined markedly from 0.191 to 0.015 per 100,000 population (AAPC = − 20.428%, 95% *CI*: − 21.076, − 19.841). Segmented regression revealed a continuous decline during 2010–2018, a more rapid reduction in 2018–2021, and a slower decrease thereafter. Incidence rates were consistently higher in males than in females, with significant declines observed in both sexes. The most pronounced reductions occurred during 2018–2021 for both sexes. Reported cases declined from 2052 in 2010 to 118 in 2023—a reduction of over 90%—with male cases consistently exceeding female cases (Tables 1, 4; Fig. 1H; Additional file 1: Table S1, Table S2, Fig.S2H).

#### Schistosomiasis

From 2010 to 2023, the incidence rate of schistosomiasis in China declined markedly from 0.339 to 0.002 per 100,000 population (AAPC = − 28.378%, 95% *CI*: − 40.688, − 15.656). A fluctuating increase occurred during 2010–2015, with notable variability between 2021 and 2023. Since 2020, the incidence has remained below 0.010 per 100,000 population. Both males and females experienced significant declines in incidence rates. Reported cases dropped sharply from 4522 in 2010 to 22 in 2023, with male cases consistently outnumbering female cases until reaching near parity in 2023 (Table 1, 4; Fig. 1I; Additional file 1: Table S1, Table S2, Fig.S2I).

### Age-gender patterns

From 2010 to 2023, the majority of zoonotic disease cases in China occurred among adolescents and adults aged 14–65 years, who accounted for 80.155% of echinococcosis, 87.740% of brucellosis, 84.476% of hemorrhagic fever, 79.194% of leptospirosis, 63.938% of rabies, 42.285% of leishmaniasis, 89.883% of anthrax, 89.250% of schistosomiasis, and 30.642% of encephalitis cases. The elderly population (over 65 years) represented a notable proportion of cases for schistosomiasis (10.154%), leptospirosis (18.935%), and rabies (21.560%), reflecting increased susceptibility with age (Additional file 1: Table S3).

Children aged 0–5 years were disproportionately affected by encephalitis (27.415%) and leishmaniasis (44.031%), while teenagers aged 5–14 years contributed significantly to encephalitis (33.059%) and rabies (9.826%) cases (Additional file 1: Table S4).

### Occupational distribution

Farmers accounted for the majority of cases across most diseases, including schistosomiasis (85.300%), brucellosis (74.260%), and hemorrhagic fever (67.898%). Herders represented 50.396% of anthrax cases, while non-institutionalized children comprised 43.569% of leishmaniasis and 31.353% of encephalitis cases. Notable proportions were also observed among students (24.847% of encephalitis; 9.094% of rabies) and retirees (3.791% of schistosomiasis) (Table 5).

**Table 5:** Occupational distribution patterns of nine zoonotic diseases in China, 2010–2023

Occupation	Echinococcosis *n* (%)	Brucellosis *n* (%)	Leptospirosis *n* (%)	Anthrax *n* (%)	Leishmaniasis *n* (%)	Encephalitis *n* (%)	Hemorrhagic fever *n* (%)	Rabies *n* (%)	Schistosomiasis *n* (%)
Fisherfolk (including boat operators)	17 (0.029)	128 (0.020)	22 (0.452)	1 (0.023)	5 (0.046)	1 (0.007)	134 (0.097)	5 (0.046)	2060 (2.468)
Nursery/daycare children	188 (0.321)	1912 (0.270)	6 (0.123)	6 (0.140)	87 (2.234)	1121 (7.382)	258 (0.187)	112 (1.037)	11 (0.013)
Healthcare professionals	172 (0.294)	2412 (0.340)	7 (0.144)	3 (0.070)	5 (0.128)	24 (0.158)	344 (0.250)	16 (0.148)	87 (0.104)
Students	2999 (5.118)	16,951 (2.360)	171 (3.516)	98 (2.284)	277 (7.112)	3773 (24.847)	5612 (4.070)	982 (9.094)	608 (0.728)
Commercial services	537 (0.916)	4023 (0.560)	89 (1.830)	16 (0.373)	37 (0.950)	83 (0.547)	2372 (1.720)	48 (0.445)	697 (0.835)
Non-institutionalized children	1042 (1.778)	7280 (1.010)	17 (0.350)	71 (1.655)	1697 (43.569)	4761 (31.353)	389 (0.282)	606 (5.612)	35 (0.042)
Farmers	25,919 (44.233)	533,631 (74.260)	3541 (72.800)	1679 (39.138)	1207 (30.988)	4086 (26.908)	93,612 (67.898)	7703 (71.337)	71,210 (85.300)
Herders	16,147 (27.556)	65,556 (9.120)	6 (0.123)	2162 (50.396)	8 (0.205)	5 (0.033)	423 (0.307)	16 (0.148)	87 (0.104)
Migrant workers	172 (0.294)	3530 (0.490)	41 (0.843)	15 (0.350)	55 (1.412)	102 (0.672)	1780 (1.291)	188 (1.741)	260 (0.311)
Retirees	1780 (3.038)	9232 (1.280)	123 (2.529)	15 (0.350)	69 (1.772)	289 (1.903)	4019 (2.915)	125 (1.158)	3165 (3.791)
Teachers	410 (0.700)	1678 (0.230)	25 (0.514)	8 (0.186)	16 (0.411)	28 (0.184)	699 (0.507)	12 (0.111)	172 (0.206)
Homemakers and the unemployed	3871 (6.606)	29,308 (4.080)	343 (7.052)	86 (2.005)	170 (4.365)	353 (2.325)	11,937 (8.658)	464 (4.297)	2323 (2.783)
Seafarers and long-distance drivers	14 (0.024)	128 (0.020)	1 (0.021)	1 (0.023)	5 (0.128)	5 (0.033)	105 (0.076)	2 (0.019)	38 (0.046)
Public venue service staff	4 (0.007)	98 (0.010)	0 (0.000)	0 (0.000)	0 (0.000)	3 (0.020)	73 (0.053)	5 (0.046)	40 (0.048)
Workers	1052 (1.795)	14,637 (2.040)	152 (3.125)	18 (0.420)	114 (2.927)	221 (1.455)	6735 (4.885)	192 (1.778)	929 (1.113)
Cadres and staff	1166 (1.99)	7729 (1.080)	59 (1.213)	11 (0.256)	50 (1.284)	81 (0.533)	2320 (1.683)	25 (0.232)	511 (0.612)
Food and beverage industry personnel	80 (0.137)	2050 (0.290)	31 (0.637)	14 (0.326)	7 (0.180)	28 (0.184)	816 (0.592)	19 (0.176)	100 (0.120)
Childcare workers and nannies	8 (0.014)	76 (0.010)	3 (0.062)	0 (0.000)	0 (0.000)	3 (0.020)	15 (0.011)	0 (0.000)	2 (0.002)
Other	2277 (3.886)	11,435 (1.590)	102 (2.097)	73 (1.702)	54 (1.386)	139 (0.915)	3610 (2.618)	165 (1.528)	676 (0.810)
Unknown	741 (1.265)	6765 (0.940)	125 (2.570)	13 (0.303)	37 (0.950)	79 (0.520)	2618 (1.899)	113 (1.046)	471 (0.564)

Data are derived from the National Notifiable Infectious Disease Reporting System of China

### Diagnostic classification

Marked differences were observed in the proportions of clinically diagnosed versus laboratory-confirmed cases across the nine zoonotic diseases. Laboratory-confirmed cases accounted for the vast majority of brucellosis (92.170%), hemorrhagic fever (77.361%), encephalitis (86.296%), and leptospirosis (54.502%) cases, indicating strong reliance on confirmatory testing for these diseases. In contrast, clinically diagnosed cases predominated in rabies (95.332%), echinococcosis (72.689%), leishmaniasis (75.327%), anthrax (83.520%), and schistosomiasis (83.664%), suggesting diagnostic challenges or limited access to laboratory confirmation in certain endemic areas (Additional file 1: Table S5).

Forecasts suggest a continued decline in the incidence of rabies (AAPC = − 2.259%, 95% *CI*: − 2.281, − 2.241) and hemorrhagic fever (AAPC = − 5.221%, 95% *CI*: − 5.235, − 5.209), with both trends being statistically significant and consistent. Leptospirosis is also expected to decrease, albeit at a slower rate (AAPC = − 0.068%, 95% *CI*: − 0.073, − 0.063). In contrast, anthrax, encephalitis, and echinococcosis are projected to exhibit modest increases, though with wide *CI*s, reflecting uncertainty in trend stability. Brucellosis is predicted to maintain a relatively stable trajectory (AAPC = 0.374%, 95% *CI*: − 0.084, 0.834), while schistosomiasis shows a markedly unstable forecast with a very high relative error and an implausibly steep rise (AAPC = 33.988%, 95% *CI*: 33.899, 34.086), likely reflecting limitations in model fit due to low baseline incidence and sparse recent data (Table 6; Additional file 1: Table S6).

**Table 6:** Forecasted incidence rate and trend changes of zoonotic diseases in China, 2024–2035

Disease	Model	Variable	Mean relative error (%) 2010–2023	Incidence rate (per 100,000 population) (95% *CI)*, 2035 year	EAPC (%) 2024–2035	AAPC (%) 2024–2035
Echinococcosis	ARIMA (1, 0, 0)	AR	0.468	0.306 (0.178, 0.498)	0.440 (0.160, 0.720)	0.440 (0.127, 0.753)
Brucellosis	ARIMA (1, 0, 0)	AR	28.514	0.334 (0.094, 0.910)	0.374 (− 0.041, 0.791)	0.374 (− 0.084, 0.834)
Leptospirosis	ARIMA (1, 0, 0)	AR	28.514	3.811 (2.267, 6.087)	− 1.435 (− 1.914, − 0.953)	− 0.068 (− 0.073, − 0.063)
Anthrax	Brown’s linear trend	*α* (level and trend)	17.527	0.035 (0.008, 0.062)	2.178 (2.127, 2.229)	1.474 (− 1.230, 5.445)
Encephalitis	ARIMA (1, 0, 0)	AR	96.652	0.084 (0.001, 0.232)	0.582 (0.202, 0.963)	0.869 (0.764, 0.985)
Rabies	Holt’s linear trend	*α* (level)	16.321	0.006 (0.001, 0.274)	− 2.255 (− 2.310, − 2.201)	− 2.259 (− 2.281, − 2.241)
		*γ* (trend)				
Hemorrhagic fever	Holt’s linear trend	*α* (level)	24.340	0.197 (0.001, 0.848)	− 5.243 (-5.286, − 5.201)	− 5.221 (− 5.235, − 5.209)
		*γ* (trend)				
Schistosomiasis	Damped Trend	*α* (level)	3256.309	0.091 (0.001, 0.142)	34.010 (33.581, 34.441)	33.988 (33.899, 34.086)
		*γ* (trend)				
		*φ* (trend damping factor)				

Data are derived from the National Notifiable Infectious Disease Reporting System of China. No suitable time series model could adequately fit the incidence rate of leishmaniasis, as reflected by poor model performance

*AAPC* average annual percent change, *AR* autoregressive, *ARIMA* autoregressive integrated moving average, *CI* confidence interval, *EAPC* estimated annual percent change

### Discrepancies between GBD 2021 estimates and nationally reported data

From 2010 to 2021, notable discrepancies were observed between incidence rates estimated by the GBD 2021 and those reported through national surveillance systems for several zoonotic diseases in China. The most striking divergence was identified in encephalitis, for which GBD estimates consistently exceeded national reports by 82.188 to 1008.733 times. For leishmaniasis, GBD-derived incidence rates were more than three-fold (ranging from 3.054 to 10.500) higher than reported values across all years. Echinococcosis also exhibited considerable differences, with fold disparities ranging from 3.611 to 7.409. In contrast, rabies showed relatively close agreement between sources, with fold differences generally near unity, although a consistent upward divergence has been noted since 2016—culminating in a nearly four-fold gap by 2021 (Table 7).

**Table 7:** Discrepancies in the incidence rate of zoonotic diseases in China: a comparative analysis of real-world surveillance data and GBD 2021 estimates, 2010–2021

Year	Echinococcosis^a^			Leishmaniasis			Encephalitis^b^			Rabies		
	GBD2021, China	Reported cases	Fold (95% *CI*)	GBD2021, China	Reported cases	Fold (95% *CI*)	GBD2021, China	Reported cases	Fold (95% *CI*)	GBD2021, China	Reported cases	Fold (95% *CI*)
	Incidence rate (per 100,000 population) (95% *UI*)	Incidence rate (per 100,000 population) (95% *UI*)		Incidence rate (per 100,000 population) (95% *UI*)	Incidence rate (per 100,000 population) (95% *UI*)		Incidence rate (per 100,000 population) (95% *UI*)	Incidence rate (per 100,000 population) (95% *UI*)		Incidence rate (per 100,000 population) (95% *UI*)	Incidence rate (per 100,000 population) (95% *UI*)	
2010	1.564 (1.165, 2.029)	0.352 (0.342, 0.362)	4.443 (3.886, 5.120)	0.143 (0.112, 0.200)	0.027 (0.024, 0.029)	5.296 (4.607, 5.985)	15.698 (14.028, 17.837)	0.191 (0.184, 0.199)	82.188 (67.560, 99.984)	0.136 (0.101, 0.160)	0.154 (0.147, 0.160)	0.883 (0.862, 0.905)
2011	1.603 (1.194, 2.078)	0.251 (0.242, 0.259)	6.386 (5.585, 7.299)	0.140 (0.113, 0.188)	0.023 (0.020, 0.025)	6.087 (5.238, 6.936)	15.586 (13.930, 17.708)	0.123 (0.117, 0.129)	126.715 (104.162, 154.152)	0.124 (0.095, 0.144)	0.143 (0.137, 0.150)	0.867 (0.843, 0.892)
2012	1.651 (1.228, 2.127)	0.259 (0.251, 0.268)	6.375 (5.548, 7.335)	0.134 (0.108, 0.173)	0.017 (0.015, 0.019)	7.882 (6.625, 9.141)	15.486 (13.830, 17.590)	0.133 (0.127, 0.139)	116.436 (95.712, 141.647)	0.105 (0.082, 0.121)	0.106 (0.100, 0.111)	0.991 (0.989, 0.992)
2013	1.698 (1.265, 2.191)	0.307 (0.298, 0.317)	5.531 (4.76, 6.378)	0.126 (0.100, 0.161)	0.012 (0.010, 0.014)	10.500 (8.534, 12.466)	15.422 (13.755, 17.514)	0.164 (0.157, 0.171)	94.037 (77.300, 114.398)	0.084 (0.065, 0.096)	0.087 (0.082, 0.092)	0.966 (0.959, 0.972)
2014	1.734 (1.297, 2.255)	0.291 (0.282, 0.300)	5.959 (5.177, 6.811)	0.119 (0.091, 0.159)	0.022 (0.019, 0.024)	5.409 (4.631, 6.187)	15.399 (13.743, 17.490)	0.063 (0.059, 0.067)	244.429 (200.924, 297.353)	0.065 (0.050, 0.076)	0.068 (0.064, 0.073)	0.956 (0.947, 0.964)
2015	1.752 (1.316, 2.285)	0.286 (0.277, 0.295)	6.126 (5.327, 7.043)	0.113 (0.082, 0.165)	0.037 (0.034, 0.041)	3.054 (2.696, 3.413)	15.452 (13.812, 17.551)	0.046 (0.042, 0.050)	335.913 (276.126, 408.646)	0.055 (0.041, 0.065)	0.059 (0.055, 0.063)	0.932 (0.919, 0.945)
2016	1.754 (1.317, 2.293)	0.386 (0.376, 0.396)	4.544 (3.965, 5.243)	0.110 (0.082, 0.154)	0.026 (0.024, 0.029)	4.231 (3.659, 4.803)	15.577 (13.906, 17.652)	0.091(0.086, 0.096)	171.176 (140.709, 208.239)	0.051 (0.037, 0.061)	0.047 (0.044, 0.051)	1.085 (1.103, 1.068)
2017	1.755 (1.321, 2.297)	0.486 (0.474, 0.497)	3.611 (3.133, 4.173)	0.110 (0.081, 0.152)	0.017 (0.015, 0.019)	6.471 (5.425, 7.516)	15.695 (13.976, 17.766)	0.084(0.079, 0.088)	186.845 (153.590, 227.301)	0.048 (0.033, 0.059)	0.037 (0.034, 0.041)	1.297 (1.365, 1.233)
2018	1.756 (1.324, 2.297)	0.354 (0.344, 0.364)	4.960 (4.344, 5.652)	0.110 (0.076, 0.168)	0.017 (0.014, 0.019)	6.471 (5.425, 7.516)	15.746 (13.983, 17.829)	0.130(0.124, 0.136)	121.123 (99.565, 147.349)	0.045 (0.030, 0.059)	0.030 (0.028, 0.033)	1.500 (1.624, 1.385)
2019	1.758 (1.324, 2.294)	0.331 (0.322, 0.341)	5.311 (4.637, 6.061)	0.108 (0.068, 0.194)	0.015 (0.013, 0.017)	7.200 (5.971, 8.431)	15.691 (13.902, 17.767)	0.031(0.028, 0.033)	506.161 (416.072, 615.757)	0.044 (0.027, 0.061)	0.021 (0.019, 0.023)	2.095 (2.422, 1.812)
2020	1.756 (1.321, 2.293)	0.263 (0.254, 0.271)	6.677 (5.848, 7.619)	0.105 (0.065, 0.195)	0.015 (0.013, 0.018)	7.000 (5.802, 8.198)	15.171 (13.438, 17.189)	0.021(0.018, 0.023)	722.429 (593.847, 878.851)	0.042 (0.023, 0.063)	0.014 (0.012, 0.016)	3.000 (3.721, 2.419)
2021	1.756 (1.315, 2.277)	0.237 (0.229, 0.245)	7.409 (6.481, 8.571)	0.106 (0.064, 0.199)	0.018 (0.015, 0.021)	5.889 (4.958, 6.819)	15.131 (13.444, 17.119)	0.015(0.013, 0.017)	1008.733 (829.194, 1227.147)	0.043 (0.023, 0.065)	0.011 (0.009, 0.013)	3.909 (5.106, 2.992)

Fold = Value _GBD2021_ /Value _reported cases_. *CI* confidence interval, *GBD* Global burden of disease, *UI* uncertainty interval

^a^The number of echinococcosis subtypes reported in National Notifiable Infectious Disease Reporting System of China exceeds that included in the GBD 2021 database. In the Chinese surveillance system, echinococcosis is classified into cystic echinococcosis (caused by *Echinococcus granulosus*) and alveolar echinococcosis (caused by *Echinococcus multilocularis*). However, the GBD 2021 database likely includes only cystic echinococcosis, without accounting for alveolar echinococcosis. Reported cases are derived from the National Notifiable Infectious Disease Reporting System of China. Moreover, the GBD 2021 database does not provide disease burden estimates for brucellosis, hemorrhagic fever, anthrax, schistosomiasis, or leptospirosis, rendering cross-database comparisons with China’s national surveillance data for these diseases infeasible

^b^In the National Notifiable Infectious Disease Reporting System of China, “encephalitis” typically refers to Japanese encephalitis, also known as epidemic Japanese encephalitis, which is an acute zoonotic infectious disease caused by the Japanese encephalitis virus. In the GBD 2021, encephalitis is classified as an independent disease entity, referring primarily to inflammation of the brain parenchyma caused by a variety of viral infections. Common etiological agents include Japanese encephalitis virus, herpes simplex virus, enteroviruses, as well as other viral pathogens such as Epstein–Barr virus and cytomegalovirus. Certain cases of acute disseminated encephalomyelitis may also be included under this category. This definition explicitly excludes non-infectious causes of encephalopathy (e.g., metabolic or toxic encephalopathies), HIV-associated encephalitis (classified under HIV/AIDS), bacterial meningitis or meningoencephalitis (which is modeled separately under the ‘meningitis and encephalitis’ group), and encephalitis caused by parasitic (e.g., amoebic) or fungal infections (typically categorized under other parasitic or fungal diseases). Thus, the GBD 2021 framework adopts a broad definition of viral encephalitis, extending beyond the narrower scope of Japanese encephalitis typically reported in China’s national notifiable infectious disease surveillance system

## Discussion

This study systematically assessed the epidemiological characteristics of nine major zoonotic diseases in China from 2010 to 2023. Significant heterogeneity was observed across diseases: the annual incidence rates of rabies, encephalitis, and schistosomiasis showed a marked declining trend, whereas echinococcosis and brucellosis exhibited persistent or fluctuating patterns. Discrepancies were noted between GBD estimates and China’s national surveillance data, with GBD 2021 substantially overestimating the disease burden in certain cases. These findings highlight the need to strengthen infectious disease surveillance systems and to develop differentiated prevention and control strategies tailored to the distinct epidemiological features of each disease, in order to effectively address the public health challenges posed by zoonotic infections in China.

## Zoonotic diseases in China

The findings revealed a significant decline in the incidence rate of rabies, schistosomiasis, and encephalitis, suggesting the effectiveness of current control strategies [37–39]. However, certain diseases, such as echinococcosis and brucellosis, exhibited fluctuating or rebounding trends in specific years, highlighting persistent challenges in achieving equitable and adequate regional disease control, as well as potential limitations in the surveillance system.

Most cases of zoonotic diseases in this study were concentrated among high-exposure occupational groups, particularly farmers and herders, who frequently interact with livestock and wildlife. This occupational pattern strongly suggests that direct or indirect contact with animals remains the primary route of transmission for many zoonotic pathogens in China. The findings underscore the pivotal role of animal reservoirs in sustaining disease circulation and highlight the need to prioritize animal health interventions as part of a comprehensive control strategy. Strengthening veterinary surveillance, promoting animal vaccination, and reducing high-risk human0-animal contact are essential to effectively reduce the burden of zoonotic diseases [40–42].

The study further identified imbalances in the proportions of clinically diagnosed versus laboratory-confirmed cases across diseases, pointing to limited pathogen confirmation capacity within China’s zoonotic disease surveillance system. This issue is particularly pronounced for emerging, re-emerging, or rare infections such as leishmaniasis and anthrax, where frontline facilities often rely on clinical judgment rather than standardized diagnostic protocols—raising the risk of misdiagnosis, underreporting, and case misclassification. Strengthening laboratory infrastructure and advancing a dual-track “sentinel-laboratory” surveillance mechanism are crucial to enhancing diagnostic accuracy and data connectivity. Standardizing and structuring surveillance data, while improving timeliness and accuracy in reporting, are essential steps toward higher-quality disease burden assessments.

As traditional high-burden diseases (e.g., rabies and schistosomiasis) come under better control, China’s zoonotic disease prevention efforts are gradually transitioning from widespread disease management to sporadic case control. During this transition, the public health system must shift from reactive risk management to proactive health governance. This includes enhancing cross-regional information sharing and joint response mechanisms, and updating legal frameworks and behavioral interventions in response to emerging risks such as increased domestic animal ownership and wildlife contact [43, 44]. Ultimately, future zoonotic disease control efforts must focus not only on curbing disease transmission but also on managing sources of infection through integrated strategies spanning ecological, behavioral, and institutional dimensions.

## Model selection and optimization

In the process of selecting and optimizing forecasting models, multiple factors should be considered, including model fit, statistical significance of parameters, and practical performance. In terms of model fit, higher *R*^2^ values and lower values of RMSE, MAPE, and NBIC generally indicate better model performance. In addition, key model parameters—such as AR, *α*, *γ*, and *φ*—must be statistically significant to ensure the model is structurally stable, parsimonious, and interpretable [45, 46]. However, model optimization should not rely solely on numerical goodness-of-fit. Real-world applicability is equally critical. Therefore, we compared the predicted values for 2010–2023 against the corresponding reported values, in order to evaluate the model’s “black-box” performance in handling historical data. This step was essential in the model selection process, providing practical insight into the model’s retrospective predictive validity [47].

This study found that certain models, despite demonstrating statistically significant parameters and acceptable goodness-of-fit metrics, exhibited substantial relative errors during empirical validation, thereby undermining their practical utility for forecasting. Furthermore, even when model parameters and fit indices fall within acceptable ranges, models whose projections deviate from established epidemiological trends should be interpreted with caution and potentially excluded from use [48, 49]. In our analysis of echinococcosis and hemorrhagic fever, both the ARIMA and Holt’s linear trend models met statistical significance criteria. Although Holt’s model yielded slightly higher relative errors compared to ARIMA, its predicted trajectories showed a declining incidence trend for both diseases—aligning more closely with recent patterns in China’s zoonotic disease control efforts, including policy implementation, sustained investments, and observable epidemiological shifts. This suggests that beyond statistical performance, alignment with real-world disease dynamics and control practices is crucial when selecting forecasting models.

Empirical evidence indicates that the incidence rate of echinococcosis has shown a sustained downward trend since 2018. This decline can be largely attributed to the strengthening of cross-sectoral governance mechanisms involving health, agriculture, and veterinary departments, as well as to the continuous increase in governmental financial investment [37]. Assuming the absence of major future epidemiological disruptions, and considering the ongoing growth in funding for infectious disease control alongside progressive improvements in public health infrastructure, it is reasonable to expect that the declining trend in echinococcosis incidence will persist in the coming years [18, 37]. Although the Holt linear trend model may carry inherent limitations in long-term forecasting, its alignment with the actual trajectory of policy interventions justified its selection in this study. This also underscores the critical role of policy adjustment variables—such as financial investment and strategic interventions—in altering the endemic patterns of infectious diseases [18, 37]. Therefore, in forecasting future trends, factors such as policy dynamics, intensity of resource allocation, and changes in epidemiological context should not only be treated as key assumptions but also serve as fundamental considerations in model selection. Incorporating these elements is essential to enhance the real-world applicability and contextual relevance of modelling in public health practice.

Modeling performance varied substantially across different zoonotic diseases, largely due to disparities in data quality, epidemiological characteristics, intervention effects, and model adaptability [25, 26]. Diseases with stable incidence patterns, comprehensive surveillance systems, and consistent intervention efforts—such as rabies—tended to yield superior model fit and forecasting accuracy. In contrast, diseases like brucellosis and leishmaniasis, influenced by ecological variability, host dynamics, behavioral factors, and surveillance sensitivity, exhibited greater temporal fluctuations and weaker time-series structures, limiting the effectiveness of traditional modeling approaches [18]. These differences underscore the necessity of disease-specific modeling strategies, and where appropriate, the incorporation of more flexible dynamic models to enhance forecast robustness and real-world utility.

## Discrepancies between GBD 2021 data and surveillance data

China’s infectious disease surveillance system demonstrates high sensitivity and data accuracy for the four zoonotic diseases analyzed in this study. Echinococcosis, leishmaniasis, and rabies are all subject to disease-specific surveillance protocols, supported by a comprehensive legal reporting framework, standardized laboratory confirmation procedures, and multi-tiered verification mechanisms. These features ensure that surveillance data are both timely and representative of real-world disease dynamics. As such, incidence rates derived from the national surveillance system are considered reliable indicators of the current epidemiological situation. However, this study found that the estimated incidence rates of echinococcosis, leishmaniasis, and rabies in recent years reported by the GBD 2021 study were generally higher than those from surveillance data. This suggests that GBD 2021 may have overestimated the disease burden. The discrepancy is likely attributable to differences in modeling logic, data sources, and the responsiveness to real-world intervention changes between the two data systems [18, 26, 50, 51].

Differences in case definitions and disease classification criteria represent a key factor contributing to the observed discrepancies. In the GBD 2021 framework, certain diseases are defined using relatively broad diagnostic categories. For example, the definition of encephalitis may encompass viral, self-limiting, and unspecified etiological forms of encephalitic syndromes. In contrast, China’s national infectious disease surveillance system typically restricts reporting to a narrower set of legally notifiable pathogens, such as Japanese encephalitis, resulting in a more specific but narrower disease classification. These inconsistencies in case definition and inclusion criteria likely contribute to the substantial differences in estimated incidence between the two data systems [18, 26, 50, 51].

The modeling approaches employed in GBD 2021—primarily DisMod-MR and Spatio-Temporal Gaussian Process Regression—rely on imputations based on geographic proximity, historical trends, and covariates when direct data are sparse or underreported. While this enhances global continuity, such extrapolation may lead to "overcorrection" in settings where high-quality surveillance data already exist, artificially inflating incidence rate estimates [18, 26, 50, 51].

GBD models also integrate diverse data sources, such as vital registration, retrospective surveys, and subnational literature. Although these enrich model inputs, some sources may suffer from bias, overestimation, or lack of standardization. In the absence of independent data verification, this can lead to upward distortion of disease burden estimates. For low-incidence or near-elimination diseases in China, such as rabies and leishmaniasis, GBD’s extrapolation to avoid “zero estimates” may further contribute to overestimation [18, 26, 50, 51].

Moreover, GBD’s core modeling structure often assumes long-term equilibrium and static parameters, limiting its ability to respond to rapid changes in disease trends resulting from real-time interventions or policy shifts. In countries like China—with well-established disease control systems, swift policy implementation, and targeted public health interventions—GBD models may fail to capture the sharp declines in incidence driven by comprehensive control measures. For instance, recent multi-sectoral investments and targeted interventions have significantly reduced the burden of rabies and echinococcosis in China, yet GBD’s trend-based projections tend to lag behind these real-world developments, leading to delayed corrections and inertia-driven overestimations [18, 26, 50, 51].

Although the GBD 2021 study offers important insights for cross-country comparisons and long-term epidemiological trend analyses, its estimates must be interpreted with caution when applied to national or subnational contexts. Particularly in informing public health decision-making—such as vaccine policy formulation, diagnostic infrastructure development, and the strategic allocation of healthcare resources—priority should be accorded to national surveillance systems that offer real-time, locally representative data. China possesses a robust foundation for constructing a domestically tailored zoonotic disease burden estimation framework. A range of high-quality national data platforms—including mortality surveillance systems, chronic disease and risk factor monitoring networks, and large-scale biomedical cohorts—has been established, alongside extensive repositories of electronic health records from clinical settings. These resources provide critical infrastructure for granular, context-specific analysis [18, 20–23, 37, 38, 44].

To further advance the accuracy and responsiveness of zoonotic disease burden assessments, there is an urgent need to promote cross-platform data integration and to develop a unified, dynamic national health data architecture that encompasses human morbidity and mortality, zoonotic reservoirs, and associated environmental and behavioral risk factors. Moreover, methodological innovations are required to reflect China’s unique epidemiological and socio-ecological realities. These include the derivation of disability weights from local population studies, customized algorithms for redistributing ill-defined causes of death, and the application of artificial intelligence to model pathogen transmission and disease progression in dynamic, multi-host ecosystems [18, 20–23, 37, 38, 44].

Importantly, the development of a nationally relevant disease burden framework extends beyond technical refinement, it necessitates sustained investment in science-policy translation mechanisms, the institutionalization of data sharing protocols, and active engagement in international scientific dialogue. Collectively, these efforts will enable China not only to generate more accurate and timely estimates of zoonotic disease burden, but also to contribute substantively to global health governance in the domain of emerging and re-emerging infectious diseases [18, 37, 38, 44].

## One Health based strategies

The study reveals persistent public health challenges. Notably, the incidence rates of brucellosis, leptospirosis, leishmaniasis, rabies, and schistosomiasis have not shown significant reductions in recent years, while anthrax has demonstrated an upward trend. These findings underscore the continued burden of zoonoses and highlight the need for more evidence-based and integrated prevention strategies [52].

It is imperative to establish a cross-sectoral, interdisciplinary surveillance and response system based on the “human-animal-environment” interface [40–42, 53]. By integrating data from China’s National Notifiable Disease Reporting System and animal epidemic surveillance platforms, a unified system should be developed that incorporates human case data, livestock infection information, and environmental exposure risks. Multi-source sentinel sites should be established in high-risk areas to enhance early warning capabilities and facilitate coordinated interventions. Precision-targeted strategies should also be implemented for high-risk populations—such as farmers, herders, young children, and older adults—including vaccination, behavioral interventions, and health education [15, 54].

Great efforts are needed to promote the integration of public health and veterinary infrastructure, particularly at the primary level in high-burden regions. Investments should be directed toward strengthening the capacity of frontline disease control personnel, veterinarians, and village health workers through joint training and coordinated outbreak response mechanisms. Based on the dynamic forecasting results from this study, resources should be prioritized for diseases and regions with rising or fluctuating incidence rates, with a focus on enhancing surveillance sensitivity and ensuring vaccine supply and accessibility [55–57].

Zoonotic disease control should be embedded within a broader framework of ecological security and health governance, serving as a key component of national strategies to mitigate the health risks associated with climate change and environmental disruption [40, 42, 53]. Policymaking should adopt a systems-based approach that fully recognizes the interdependence of human, animal, and environmental health. The “One Health” paradigm should be institutionalized within national development planning, supported by scientific modeling, data integration, and technological innovation to strengthen the scientific rigor, foresight, and sustainability of zoonotic disease prevention and control efforts [55–57].

This study has several methodological and surveillance-related limitations that warrant consideration. First, although various time series models (e.g., ARIMA, Holt, Brown, and Damped Trend) were applied to project disease incidence, the overall model fit was suboptimal for certain diseases, such as leishmaniasis and brucellosis, with low *R*^2^ values or high MAPE, suggesting limited predictive validity. Moreover, seasonal patterns, external covariates (e.g., climatic or policy factors), and potential structural breakpoints were not incorporated into the modeling process [25, 26]. Second, the stratified analyses by age, sex, and occupation were descriptive in nature, lacking multi-variable statistical adjustment to control for confounding or assess independent risk associations [18, 19, 25, 26]. Third, discrepancies between the GBD 2021 estimates and national surveillance data were identified, but the study did not fully explore the potential sources of these divergences, such as differences in case definitions, diagnostic capacities, or data completeness [18]. Finally, the reliance on national surveillance data may be subject to underreporting and diagnostic misclassification, particularly for diseases with low laboratory confirmation rates. These limitations underscore the need for strengthened surveillance sensitivity, enhanced model calibration, and integration of real-world and modeled data for more accurate burden estimation and policy guidance.

## Conclusions

This study found that while the incidence of several major zoonotic diseases in China—such as rabies, encephalitis, and schistosomiasis—has declined markedly in recent years due to enhanced multi-sectoral control efforts, others, including echinococcosis and brucellosis, continue to display significant annual fluctuations. These diseases exhibit recurrent, insidious, and geographically clustered transmission patterns, underscoring ongoing challenges in their control and the need for sustained vigilance. To address this complexity, tailored, disease-specific strategies should be formulated based on distinct transmission dynamics and the exposure profiles of high-risk populations. Such strategies should enable stratified interventions, efficient resource allocation, and dynamic adaptation of control measures. Ultimately, establishing a zoonotic disease control system grounded in the One Health framework is critical for coordinated management of human, animal, and environmental health. This integrated approach is essential for interrupting cross-species transmission at its source and mitigating the long-term public health threats posed by zoonoses.

## Supplementary Information

Additional file 1: Fig.S1. Vertical Reporting Structure, Chinese Infectious Diseases Reporting System. Table S1. Incidence rates of nine common zoonotic diseases in China, 2010–2023. Table S2. Number of reported cases of nine common zoonotic diseases in China, 2010–2023. Fig. S2. Age–sex patterns and temporal trends of nine zoonotic diseases in the China, 2010–2023. Table S3. Gender-specific distribution characteristics of nine zoonotic infectious diseases in China, 2010–2023. Table S4. Age-specific distribution characteristics of nine zoonotic infectious diseases in China, 2010–2023. Table S5. Clinically diagnosed and laboratory-confirmed cases of nine zoonotic infectious diseases in China, 2010–2023. Table S6. Modeling the incidence rates of nine common zoonotic diseases in China, 2024–2035. Table S7. Projected values and temporal trends of zoonotic diseases in China, 2024–2035.

## Data Availability

The data is confidential. To gain access, data requesters will need to sign a data access agreement, upon reasonable request. Proposals should be directed to the corresponding author (Email: zhengcj@chinacdc.cn).

## Abbreviations

APC: Annual percentage change
AAPC: Average annual percent change
AR: Autoregressive
ARIMA: Autoregressive integrated moving average
CDC: Center for Disease Control and Prevention
*CI*: Confidence interval
COVID-19: Coronavirus disease 2019
EAPC: Estimation of the annual percentage change
GBD: Global Burden of Disease
MA: Moving average
MAPE: Mean absolute percentage error
MRE: Mean relative error
MSE: Mean squared error
NBIC: Normalized Bayesian Information Criterion
NNIDRS: National Notifiable Infectious Disease Reporting System
RMSE: Root mean square error
SARS: Severe acute respiratory syndrome
*UI*: Uncertainty intervals
