# Supplementary material for: Zoonotic diseases in China: epidemiological trends, incidence forecasting, and comparative analysis between real-world surveillance data and Global Burden of Disease 2021 estimates
Source: Infect Dis Poverty. 2025 Jul 4;14:60. doi: 10.1186/s40249-025-01335-3 (PMC12231708; doi:10.1186/s40249-025-01335-3)
Supplement: Supplementary file 1 — Additional file 1: Fig.S1. Vertical Reporting Structure, Chinese Infectious Diseases Reporting System. Table S1. Incidence rates of nine common zoonotic diseases in China, 2010–2023. Table S2. Number of reported cases of nine common zoonotic diseases in China, 2010–2023. Fig. S2. Age–sex patterns and temporal trends of nine zoonotic diseases in the China, 2010–2023. Table S3. Gender-specific distribution characteristics of nine zoonotic infectious diseases in China, 2010–2023. Table S4. Age-specific distribution characteristics of nine zoonotic infectious diseases in China, 2010–2023. Table S5. Clinically diagnosed and laboratory-confirmed cases of nine zoonotic infectious diseases in China, 2010–2023. Table S6. Modeling the incidence rates of nine common zoonotic diseases in China, 2024–2035. Table S7. Projected values and temporal trends of zoonotic diseases in China, 2024–2035. [file 40249_2025_1335_MOESM1_ESM.docx]

**Additional file 1**

**Zoonotic diseases in China: epidemiological trends, incidence forecasting, and comparative analysis between real-world surveillance data and Global Burden of Disease 2021 estimates**

Yun-Fei Zhang^1†^, Shi-Zhu Li^2†^, Shi-Wen Wang^3†^, Di Mu^4^, Xi Chen^4^, Sheng Zhou^4^, Hai-Jian Zhou^1^, Tian Qin^1^, Qin Liu^2^, Shan Lv^2^, Yan Lu^2^, Ji-Chun Wang^4^, Yu Qin^4^, Guo-Bing Yang^5^, Yong-Jun Li^5^, Jian-Yun Sun^5^, Xiao-Nong Zhou^2^, Mai-Geng Zhou^6^, Can-Jun Zheng^3*^, Biao Kan^1*^, and Shun-Xian Zhang^7*^

^†^ contributed equally to this work.

* Corresponding author.

Can-Jun Zheng, [zhengcj@chinacdc.cn](mailto:18817338863@126.com);)

Biao Kan, kanbiao@icdc.cn

Shun-Xian Zhang,  [zhangshunxian110@163.com](mailto:18817338863@126.com);)

^1^ National Key Laboratory of Intelligent Tracking and Forecasting for Infectious Diseases, National Institute for Communicable Disease Control and Prevention, Chinese Center for Disease Control and Prevention, Beijing 102206, China.

^2^ National Key Laboratory of Intelligent Tracking and Forecasting for Infectious Diseases, NHC Key Laboratory of Parasite and Vector Biology, WHO Collaborating Centre for Tropical Diseases, National Center for International Research on Tropical Diseases, National Institute of Parasitic Diseases of Chinese Center for Disease Control and Prevention, Shanghai 200025, China.

^3^ National Key Laboratory of Intelligent Tracking and Forecasting for Infectious Diseases, National institute for viral disease control and prevention, Chinese Center for Disease Control and Prevention, Beijing 102206, China.

^4^ National Key Laboratory of Intelligent Tracking and Forecasting for Infectious Diseases, Chinese Center for Disease Control and Prevention, Beijing 102206, China.

^5^ Gansu Provincial Center for Disease Control and Prevention, Gansu Provincial Academy of Preventive Medicine, Lanzhou 730000, Gansu, China.

^6^ National Center for Chronic and Noncommunicable Disease Control and Prevention, Chinese Center for Disease Control and Prevention, Beijing 100050, China.

^7^ Longhua Hospital, Shanghai University of Traditional Chinese Medicine, Shanghai 200032, China.

**Items**

Forecasting models

Fig.S1: Vertical Reporting Structure, Chinese Infectious Diseases Reporting System. Table S1: Incidence rates of nine common zoonotic diseases in Chinese mainland, 2010–2023.

Table S2: Number of reported cases of nine common zoonotic diseases in Chinese mainland, 2010–2023.

Fig. S2: Age–sex patterns and temporal trends of nine zoonotic diseases in the Chinese Mainland, 2010–2023.

Table S3: Gender-specific distribution characteristics of nine zoonotic infectious diseases in Chinese mainland, 2010–2023.

Table S4: Age-specific distribution characteristics of nine zoonotic infectious diseases in Chinese mainland, 2010–2023.

Table S5: Clinically diagnosed and laboratory-confirmed cases of nine zoonotic infectious diseases in Chinese mainland, 2010–2023.

Table S6: Modeling the incidence rates of nine common zoonotic diseases in Chinese mainland, 2024–2035.

Table S7: Projected values and temporal trends of zoonotic diseases in Chinese mainland, 2024–2035

**Forecasting models**

*ARIMA*

The incidence rates of the nine zoonotic diseases from 2010 to 2023 were used to forecast their trends from 2023 to 2035 by applying the ARIMA model. An ARIMA time series model was constructed, and the stationarity of the data was assessed using autocorrelation function (ACF) and partial autocorrelation function (PACF) plots. For non-stationary sequences, data transformations such as natural logarithmic transformation, square root transformation, and differencing were applied to stabilize the variance and remove trends[1-4].

ARIMA model identification: Based on the ACF and PACF plots, and preliminary fitting results from R software, tentative model structures were identified. The ARIMA (p,d,q) model was selected as the initial framework. According to established practice, the orders p and q were restricted to no more than 3, and the differencing order d was limited to a maximum of 2. Consequently, model parameters p, q, P, and Q were evaluated sequentially from lower to higher orders to determine the optimal structure [1,4].

ARIMA model diagnostics and parameter evaluation: For ARIMA models, parameters were estimated using the nonlinear least squares method. The model residuals were then subjected to the Ljung–Box Q test to assess whether they constituted white noise, which would indicate the model's adequacy. Candidate models were evaluated based on several diagnostic criteria, including: (1) non-significant Ljung–Box Q statistics (P > 0.05), with lower Q values indicating better model fit; (2) parsimony of the model, favoring lower orders of p, d, and q; (3) statistical significance of autoregressive (AR) and moving average (MA) terms (*P* < 0.05); (4) higher stationary R-squared (indicating better model stability); (5) lower values of the normalized Bayesian Information Criterion (NBIC); and (6) smaller root mean square error (RMSE) values[1 4].

*Exponential smoothing models*

The incidence rates of the nine zoonotic diseases from 2010 to 2023 were used to forecast trends from 2023 to 2035 by applying exponential smoothing models, including Holt’s linear trend, Brown’s linear trend, and the damped trend model.

Holt’s linear trend: it extends simple exponential smoothing by incorporating a second component to account for linear trends in the data. It uses two smoothing parameters—α (alpha) for the level component and β (beta) for the trend component—to iteratively update both the current level $l_{t}$ and trend $b{}_{t}$​ of the time series[1, 4]**.** The model generates the forecast $\mathop{y}\limits^{\wedge} _{t+h}=l_{t}+h\times b_{t}$by extrapolating the level and trend h steps into the future. This dual-parameter structure allows the model to dynamically respond to gradual shifts in trend direction or intensity, making it particularly effective for short- and medium-term forecasting when data exhibit persistent upward or downward movement.

**Brown’s linear trend model: it also known as double exponential smoothing, captures trend dynamics by applying exponential smoothing twice—first to the original series and then to the smoothed values. Unlike Holt’s model, Brown’s method employs a single smoothing parameter, α (alpha), which simultaneously updates both the level and the trend. The trend is inferred through the difference between the single and double smoothed values**[5 6]**. Its forecast equation follows the form** $\mathop{y}\limits^{\wedge} _{t+h}=a_{t}+b_{t}\times h_{t}$, w**here $a_{t}$and$b_{t}$represent the estimated level and trend, respectively. Although simpler in structure, Brown’s model assumes a constant linear trend, which may reduce adaptability under evolving or nonlinear data patterns.**

**Damped trend model: is a modification of Holt’s method that incorporates a damping parameter, φ (phi), to reduce the contribution of the trend over time, thereby producing more conservative long-term forecasts. It retains the two core smoothing parameters—α (alpha) and β (beta)—to update the level and trend components, but modifies the extrapolation with the equation**[5]**,** $\mathop{y}\limits^{\wedge} _{t+h}=(l_{t}+\phi+\phi^{2}+\bullet\bullet\bullet+\phi^{h-1})\times b_{t}$.This cumulative damping term effectively limits the influence of $b_{t}$ as increases. The inclusion of φ (0 < φ < 1) addresses the unrealistic assumption of an infinite linear trend and enhances forecast stability in systems where eventual trend attenuation is expected.

Similarly, for the exponential smoothing models—namely Holt’s linear trend (parameters: alpha and gamma), Brown’s linear trend (alpha), and the damped trend model (alpha, gamma, and phi)—model selection was guided by the requirement that at least one parameter be statistically significant (*P* < 0.05). Additional selection criteria included higher stationary R-squared, lower NBIC, and lower RMSE values to ensure optimal predictive performance[6, 7].

*Model optimization and practical application*

The optimal model for each disease was then employed to project the incidence rate of the nine zoonotic diseases in Chinese mainland for the years 2024 to 2035[8, 9]. After ensuring that candidate models meet fundamental assumptions, the optimal model was selected based on minimizing the relative error between predicted and actual monthly case counts from 2010 to 2023. Each candidate model was used to forecast monthly reported cases for each disease during this period. The model yielding the smallest mean relative error between predicted and observed case counts was chosen as the optimal model.

[1]. Gao, S., et al., Global Trends in Incidence and Mortality Rates of Endometrial Cancer Among Individuals Aged 55 years and Above From 1990 to 2021: An Analysis of the Global Burden of Disease. Int J Womens Health, 2025. 17: p. 651-662.

[2]. Chen, Y., et al., Global burden of HIV-negative multidrug- and extensively drug-resistant tuberculosis based on Global Burden of Disease Study 2021. Science in One Health, 2024. 3: p. 100072.

[3]. Zheng, J., et al., Global burden of malaria and neglected tropical diseases in children and adolescents, 1990-2019: a population-based, cross-sectional study. J R Soc Med, 2025: p. 1410768251321572.

[4]. Zhang, S., et al., Global burden of low vision and blindness due to age-related macular degeneration from 1990 to 2021 and projections for 2050. BMC Public Health, 2024. 24(1): p. 3510.

[5]. Lopes Antunes, A.C. and D. Jensen, Comparison of time-series models for monitoring temporal trends in endemic diseases sero-prevalence: lessons from porcine reproductive and respiratory syndrome in Danish swine herds. BMC Vet Res, 2019. 15(1): p. 231.

[6]. Zhao, D., Research of Combined ES-BP Model in Predicting Syphilis Incidence 1982-2020 in Mainland China. Iran J Public Health, 2023. 52(10): p. 2063-2072.

[7]. Saulnier, G.E., J.C. Castro and C.B. Cook, Impact of measurement error on predicting population-based inpatient glucose control. Future Sci OA, 2019. 5(5): p. FSO388.

[8]. Boracchini, R., et al., Exploring different modelling approaches to forecast the community acute respiratory infections burden in children: an Italian epidemiological time series study. BMC Public Health, 2025. 25(1): p. 810.

[9]. Shen, Z. and H. Luo, The impact of schistosomiasis on the Global Disease Burden: a systematic analysis based on the 2021 Global Burden of Disease study. Parasite, 2025. 32: p. 12.


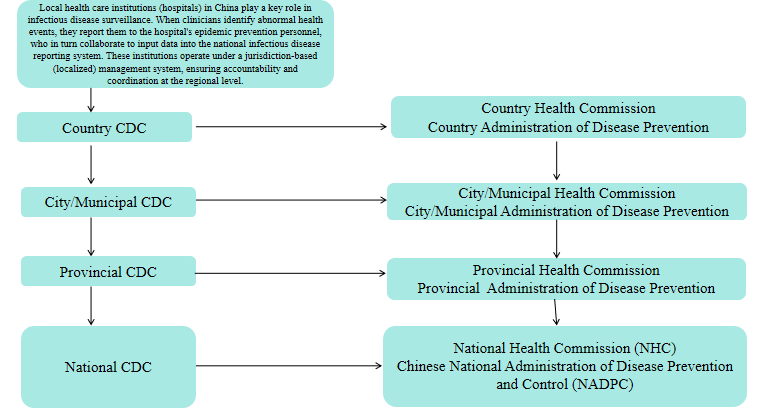


**Fig. S1.** Vertical Reporting Structure, Chinese Infectious Diseases Reporting System (Figure from China Information System for Disease Control and Prevention).

**Table S1**: Incidence rates of nine common zoonotic diseases in Chinese mainland, 2010–2023

| Year | Echinococcosis Incidence rate | | | Brucellosis Incidence rate | | | Hemorrhagic fever Incidence rate | | |
| --- | --- | --- | --- | --- | --- | --- | --- | --- | --- |
|  | Both gender (per 100,000 population) | Male (per 100,000 population) | Female (per 100,000 population) | Both gender (per 100,000 population) | Male (per 100,000 population ) | Female (per 100,000 population ) | Both gender (per 100,000 population ) | Male (per 100,000 population ) | Female (per 100,000 population) |
| 2010 | 0.352(0.342, 0.362) | 0.346(0.332, 0.36) | 0.359(0.344, 0.374) | 3.044(3.014, 3.073) | 4.345(4.295, 4.394) | 1.675(1.644, 1.707) | 0.706(0.692, 0.72) | 1.05(1.026, 1.074) | 0.344(0.33, 0.358) |
| 2011 | 0.251(0.242, 0.259) | 0.237(0.225, 0.248) | 0.265(0.253, 0.278) | 2.99(2.961, 3.019) | 4.354(4.305, 4.404) | 1.554(1.524, 1.584) | 0.804(0.789, 0.82) | 1.18(1.154, 1.205) | 0.41(0.394, 0.425) |
| 2012 | 0.259(0.251, 0.268) | 0.25(0.238, 0.262) | 0.269(0.257, 0.282) | 3.05(3.021, 3.08) | 4.477(4.427, 4.527) | 1.549(1.519, 1.579) | 0.985(0.968, 1.001) | 1.446(1.417, 1.474) | 0.5(0.483, 0.517) |
| 2013 | 0.307(0.298, 0.317) | 0.276(0.264, 0.289) | 0.34(0.326, 0.354) | 3.332(3.301, 3.363) | 4.801(4.749, 4.852) | 1.788(1.756, 1.82) | 0.952(0.936, 0.968) | 1.355(1.327, 1.382) | 0.529(0.511, 0.546) |
| 2014 | 0.291(0.282, 0.300) | 0.26(0.248, 0.272) | 0.324(0.31, 0.338) | 4.371(4.336, 4.406) | 6.275(6.216, 6.334) | 2.363(2.326, 2.401) | 0.854(0.839, 0.87) | 1.219(1.193, 1.245) | 0.469(0.452, 0.485) |
| 2015 | 0.286(0.277, 0.295) | 0.257(0.246, 0.269) | 0.315(0.302, 0.329) | 4.344(4.309, 4.379) | 6.159(6.101, 6.218) | 2.437(2.399, 2.474) | 0.754(0.74, 0.769) | 1.091(1.067, 1.116) | 0.401(0.386, 0.416) |
| 2016 | 0.386(0.376, 0.396) | 0.357(0.343, 0.371) | 0.417(0.401, 0.432) | 3.569(3.537, 3.6) | 5.074(5.022, 5.127) | 1.987(1.953, 2.021) | 0.647(0.634, 0.661) | 0.936(0.914, 0.959) | 0.344(0.329, 0.358) |
| 2017 | 0.486(0.474, 0.497) | 0.447(0.431, 0.462) | 0.527(0.509, 0.544) | 2.906(2.877, 2.934) | 4.125(4.078, 4.173) | 1.631(1.601, 1.662) | 0.81(0.795, 0.825) | 1.171(1.145, 1.196) | 0.433(0.417, 0.449) |
| 2018 | 0.354(0.344, 0.364) | 0.335(0.322, 0.349) | 0.373(0.359, 0.388) | 2.842(2.814, 2.87) | 3.964(3.918, 4.011) | 1.671(1.64, 1.702) | 0.863(0.848, 0.879) | 1.231(1.205, 1.257) | 0.48(0.463, 0.496) |
| 2019 | 0.331(0.322, 0.341) | 0.319(0.306, 0.332) | 0.344(0.33, 0.358) | 3.277(3.247, 3.307) | 4.599(4.549, 4.649) | 1.896(1.863, 1.929) | 0.695(0.681, 0.708) | 0.99(0.967, 1.013) | 0.386(0.372, 0.401) |
| 2020 | 0.263(0.254, 0.271) | 0.247(0.235, 0.258) | 0.279(0.267, 0.292) | 3.515(3.484, 3.546) | 4.873(4.822, 4.924) | 2.097(2.063, 2.132) | 0.581(0.569, 0.594) | 0.825(0.804, 0.846) | 0.327(0.313, 0.34) |
| 2021 | 0.237(0.229, 0.245) | 0.224(0.213, 0.235) | 0.249(0.237, 0.261) | 5.11(5.072, 5.147) | 7.160(7.098, 7.222) | 2.941(2.9, 2.981) | 0.647(0.634, 0.661) | 0.915(0.893, 0.937) | 0.364(0.35, 0.379) |
| 2022 | 0.194(0.187, 0.202) | 0.177(0.167, 0.187) | 0.212(0.201, 0.223) | 4.861(4.824, 4.897) | 6.759(6.699, 6.819) | 2.865(2.825, 2.905) | 0.38(0.37, 0.39) | 0.534(0.517, 0.55) | 0.219(0.207, 0.23) |
| 2023 | 0.265(0.256, 0.273) | 0.241(0.23, 0.253) | 0.29(0.277, 0.302) | 4.894(4.858, 4.931) | 6.754(6.694, 6.814) | 2.952(2.912, 2.993) | 0.373(0.363, 0.383) | 0.527(0.51, 0.544) | 0.212(0.201, 0.223) |
|  |  |  |  |  |  |  |  |  |  |
| Year | Leptospirosis Incidence rate | | | Leishmaniasis Incidence rate | | | Anthrax Incidence rate | | |
|  | Both gender (per 100,000 population) | Male (per 100,000 population) | Female (per 100,000 population) | Both gender (per 100,000) | Male (per 100,000 population) | Female (per 100,000 population) | Both gender (per 100,000 population) | Male (per 100,000 population) | Female (per 100,000 population) |
| 2010 | 0.047(0.047, 0.055) | 0.062(0.062, 0.075) | 0.028(0.028, 0.037) | 0.027(0.024, 0.029) | 0.032(0.028, 0.037) | 0.021(0.017, 0.024) | 0.022(0.019, 0.024) | 0.03(0.026, 0.034) | 0.013(0.01, 0.016) |
| 2011 | 0.027(0.027, 0.033) | 0.035(0.035, 0.044) | 0.016(0.016, 0.022) | 0.023(0.02, 0.025) | 0.029(0.025, 0.033) | 0.017(0.013, 0.02) | 0.023(0.02, 0.026) | 0.033(0.029, 0.038) | 0.012(0.009, 0.015) |
| 2012 | 0.03(0.03, 0.036) | 0.039(0.039, 0.049) | 0.018(0.018, 0.025) | 0.017(0.015, 0.019) | 0.021(0.017, 0.024) | 0.012(0.01, 0.015) | 0.018(0.015, 0.02) | 0.024(0.02, 0.028) | 0.011(0.009, 0.014) |
| 2013 | 0.023(0.023, 0.029) | 0.028(0.028, 0.037) | 0.016(0.016, 0.023) | 0.012(0.01, 0.014) | 0.016(0.013, 0.019) | 0.008(0.006, 0.011) | 0.014(0.012, 0.016) | 0.021(0.018, 0.025) | 0.007(0.005, 0.009) |
| 2014 | 0.034(0.034, 0.04) | 0.039(0.039, 0.049) | 0.025(0.025, 0.034) | 0.022(0.019, 0.024) | 0.023(0.02, 0.027) | 0.02(0.017, 0.023) | 0.018(0.016, 0.02) | 0.025(0.022, 0.029) | 0.011(0.008, 0.013) |
| 2015 | 0.023(0.023, 0.029) | 0.03(0.03, 0.039) | 0.014(0.014, 0.021) | 0.037(0.034, 0.041) | 0.043(0.038, 0.048) | 0.031(0.027, 0.035) | 0.021(0.019, 0.024) | 0.031(0.027, 0.035) | 0.011(0.009, 0.014) |
| 2016 | 0.023(0.023, 0.028) | 0.025(0.025, 0.033) | 0.018(0.018, 0.025) | 0.026(0.024, 0.029) | 0.029(0.025, 0.033) | 0.023(0.02, 0.027) | 0.027(0.024, 0.03) | 0.039(0.034, 0.044) | 0.015(0.012, 0.018) |
| 2017 | 0.013(0.013, 0.018) | 0.02(0.02, 0.027) | 0.005(0.005, 0.009) | 0.017(0.015, 0.019) | 0.022(0.019, 0.026) | 0.011(0.009, 0.014) | 0.023(0.021, 0.026) | 0.034(0.03, 0.038) | 0.012(0.009, 0.014) |
| 2018 | 0.009(0.009, 0.013) | 0.015(0.015, 0.021) | 0.003(0.003, 0.006) | 0.017(0.014, 0.019) | 0.02(0.017, 0.023) | 0.013(0.01, 0.016) | 0.024(0.021, 0.027) | 0.035(0.03, 0.039) | 0.013(0.01, 0.015) |
| 2019 | 0.014(0.014, 0.018) | 0.021(0.021, 0.028) | 0.005(0.005, 0.009) | 0.015(0.013, 0.017) | 0.02(0.017, 0.023) | 0.01(0.007, 0.012) | 0.021(0.019, 0.024) | 0.033(0.028, 0.037) | 0.01(0.007, 0.012) |
| 2020 | 0.019(0.019, 0.024) | 0.028(0.028, 0.036) | 0.008(0.008, 0.013) | 0.015(0.013, 0.018) | 0.021(0.018, 0.024) | 0.01(0.007, 0.012) | 0.016(0.014, 0.018) | 0.019(0.016, 0.023) | 0.012(0.009, 0.015) |
| 2021 | 0.026(0.026, 0.031) | 0.039(0.039, 0.049) | 0.01(0.01, 0.015) | 0.018(0.015, 0.02) | 0.024(0.02, 0.027) | 0.011(0.009, 0.014) | 0.028(0.025, 0.03) | 0.041(0.036, 0.046) | 0.014(0.011, 0.016) |
| 2022 | 0.012(0.012, 0.015) | 0.018(0.018, 0.024) | 0.004(0.004, 0.007) | 0.017(0.015, 0.019) | 0.024(0.021, 0.028) | 0.01(0.008, 0.012) | 0.025(0.022, 0.027) | 0.034(0.03, 0.038) | 0.015(0.012, 0.018) |
| 2023 | 0.019(0.019, 0.024) | 0.029(0.029, 0.037) | 0.007(0.007, 0.012) | 0.021(0.019, 0.024) | 0.03(0.026, 0.034) | 0.012(0.009, 0.014) | 0.031(0.028, 0.034) | 0.045(0.04, 0.05) | 0.016(0.013, 0.019) |
|  |  |  |  |  |  |  |  |  |  |
| Year | Schistosomiasis Incidence rate | | | Encephalitis Incidence rate | | | Rabies Incidence rate | | |
|  | Both gender (per 100,000 population) | Male (per 100,000 population) | Female (per 100,000 population) | Both gender (per 100,000 population) | Male (per 100,000 population) | Female (per 100,000 population) | Both gender (per 100,000 population) | Male (per 100,000 population) | Female (per 100,000 population) |
| 2010 | 0.339(0.329, 0.349) | 0.412(0.397, 0.428) | 0.261(0.249, 0.274) | 0.191(0.184, 0.199) | 0.223(0.212, 0.234) | 0.158(0.148, 0.168) | 0.191(0.184, 0.199) | 0.223(0.212, 0.234) | 0.158(0.148, 0.168) |
| 2011 | 0.349(0.339, 0.359) | 0.406(0.391, 0.421) | 0.289(0.276, 0.302) | 0.123(0.117, 0.129) | 0.139(0.13, 0.148) | 0.106(0.098, 0.114) | 0.123(0.117, 0.129) | 0.139(0.13, 0.148) | 0.106(0.098, 0.114) |
| 2012 | 0.372(0.361, 0.382) | 0.434(0.418, 0.449) | 0.306(0.293, 0.319) | 0.133(0.127, 0.139) | 0.155(0.146, 0.165) | 0.109(0.101, 0.117) | 0.133(0.127, 0.139) | 0.155(0.146, 0.165) | 0.109(0.101, 0.117) |
| 2013 | 0.429(0.418, 0.44) | 0.493(0.476, 0.509) | 0.362(0.347, 0.376) | 0.164(0.157, 0.171) | 0.175(0.166, 0.185) | 0.153(0.143, 0.162) | 0.164(0.157, 0.171) | 0.175(0.166, 0.185) | 0.153(0.143, 0.162) |
| 2014 | 0.333(0.324, 0.343) | 0.383(0.368, 0.397) | 0.281(0.268, 0.294) | 0.063(0.059, 0.067) | 0.072(0.066, 0.079) | 0.053(0.048, 0.059) | 0.063(0.059, 0.067) | 0.072(0.066, 0.079) | 0.053(0.048, 0.059) |
| 2015 | 3.229(3.199, 3.259) | 3.872(3.826, 3.918) | 2.554(2.516, 2.592) | 0.046(0.042, 0.05) | 0.05(0.045, 0.056) | 0.042(0.037, 0.046) | 0.046(0.042, 0.05) | 0.05(0.045, 0.056) | 0.042(0.037, 0.046) |
| 2016 | 0.556(0.543, 0.568) | 0.81(0.789, 0.831) | 0.289(0.276, 0.302) | 0.091(0.086, 0.096) | 0.094(0.087, 0.101) | 0.089(0.082, 0.096) | 0.091(0.086, 0.096) | 0.094(0.087, 0.101) | 0.089(0.082, 0.096) |
| 2017 | 0.489(0.477, 0.5) | 0.719(0.699, 0.738) | 0.248(0.236, 0.26) | 0.084(0.079, 0.088) | 0.091(0.084, 0.098) | 0.076(0.069, 0.082) | 0.084(0.079, 0.088) | 0.091(0.084, 0.098) | 0.076(0.069, 0.082) |
| 2018 | 0.016(0.014, 0.018) | 0.022(0.019, 0.025) | 0.009(0.007, 0.012) | 0.13(0.124, 0.136) | 0.132(0.124, 0.141) | 0.127(0.119, 0.136) | 0.13(0.124, 0.136) | 0.132(0.124, 0.141) | 0.127(0.119, 0.136) |
| 2019 | 0.014(0.012, 0.016) | 0.018(0.014, 0.021) | 0.01(0.008, 0.013) | 0.031(0.028, 0.033) | 0.037(0.033, 0.042) | 0.024(0.02, 0.027) | 0.031(0.028, 0.033) | 0.037(0.033, 0.042) | 0.024(0.02, 0.027) |
| 2020 | 0.004(0.003, 0.005) | 0.005(0.003, 0.007) | 0.002(0.001, 0.003) | 0.021(0.018, 0.023) | 0.026(0.022, 0.03) | 0.015(0.012, 0.018) | 0.021(0.018, 0.023) | 0.026(0.022, 0.03) | 0.015(0.012, 0.018) |
| 2021 | 0.001(0.001, 0.002) | 0.001(0.001, 0.002) | 0.001(0, 0.001) | 0.015(0.013, 0.017) | 0.017(0.014, 0.02) | 0.012(0.01, 0.015) | 0.015(0.013, 0.017) | 0.017(0.014, 0.02) | 0.012(0.01, 0.015) |
| 2022 | 0.007(0.005, 0.008) | 0.007(0.005, 0.009) | 0.007(0.005, 0.008) | 0.01(0.009, 0.012) | 0.011(0.008, 0.013) | 0.01(0.008, 0.013) | 0.01(0.009, 0.012) | 0.011(0.008, 0.013) | 0.01(0.008, 0.013) |
| 2023 | 0.002(0.001, 0.002) | 0.001(0.001, 0.002) | 0.002(0.001, 0.003) | 0.015(0.013, 0.017) | 0.016(0.013, 0.019) | 0.013(0.01, 0.015) | 0.015(0.013, 0.017) | 0.016(0.013, 0.019) | 0.013(0.01, 0.015) |

Notes: In the National Notifiable Infectious Disease Reporting System (NNIDRS) of China, "Encephalitis" typically refers to Japanese encephalitis (JE), also known as epidemic Japanese encephalitis, which is an acute zoonotic infectious disease caused by the Japanese encephalitis virus (JEV). The disease is primarily transmitted by mosquitoes and is classified as a Category B notifiable infectious disease under Chinese law, requiring mandatory reporting and surveillance.

**Table S2**: Number of reported cases of nine common zoonotic diseases in Chinese mainland, 2010–2023

| Year | Echinococcosis | | | Brucellosis | | | Hemorrhagic fever | | |
| --- | --- | --- | --- | --- | --- | --- | --- | --- | --- |
|  | Both gender (persons) | Male (persons) | Female (persons) | Both gender (persons) | Male (persons) | Female (persons) | Both gender (persons) | Male (persons) | Female (persons) |
| 2010 | 4702 | 2367 | 2335 | 40627 | 29729 | 10898 | 9423 | 7186 | 2237 |
| 2011 | 3363 | 1629 | 1734 | 40092 | 29936 | 10156 | 10787 | 8109 | 2678 |
| 2012 | 3496 | 1728 | 1768 | 41097 | 30924 | 10173 | 13267 | 9984 | 3283 |
| 2013 | 4162 | 1917 | 2245 | 45117 | 33315 | 11802 | 12891 | 9400 | 3491 |
| 2014 | 3947 | 1809 | 2138 | 59237 | 43648 | 15589 | 11574 | 8482 | 3092 |
| 2015 | 3892 | 1797 | 2095 | 59182 | 42990 | 16192 | 10278 | 7615 | 2663 |
| 2016 | 5291 | 2505 | 2786 | 48918 | 35631 | 13287 | 8872 | 6575 | 2297 |
| 2017 | 6703 | 3150 | 3553 | 40097 | 29095 | 11002 | 11179 | 8257 | 2922 |
| 2018 | 4915 | 2379 | 2536 | 39480 | 28124 | 11356 | 11994 | 8733 | 3261 |
| 2019 | 4625 | 2277 | 2348 | 45764 | 32814 | 12950 | 9701 | 7061 | 2640 |
| 2020 | 3687 | 1770 | 1917 | 49349 | 34947 | 14402 | 8163 | 5919 | 2244 |
| 2021 | 3337 | 1623 | 1714 | 72036 | 51790 | 20246 | 9125 | 6617 | 2508 |
| 2022 | 2740 | 1277 | 1463 | 68564 | 48806 | 19758 | 5360 | 3853 | 1507 |
| 2023 | 3734 | 1738 | 1996 | 68999 | 48648 | 20351 | 5257 | 3796 | 1461 |
|  |  |  |  |  |  |  |  |  |  |
| year | Leptospirosis | Leptospirosis | Leptospirosis | Leishmaniasis | Leishmaniasis | Leishmaniasis | Anthrax | Anthrax | Anthrax |
|  | Both gender (persons) | Male (persons) | Female (persons) | Both gender (persons) | Male (persons) | Female (persons) | Both gender (persons) | Male (persons) | Female (persons) |
| 2010 | 679 | 468 | 211 | 354 | 221 | 133 | 292 | 206 | 86 |
| 2011 | 397 | 273 | 124 | 307 | 199 | 108 | 308 | 230 | 78 |
| 2012 | 444 | 301 | 143 | 225 | 144 | 81 | 238 | 165 | 73 |
| 2013 | 355 | 225 | 130 | 164 | 109 | 55 | 193 | 148 | 45 |
| 2014 | 499 | 305 | 194 | 294 | 162 | 132 | 247 | 177 | 70 |
| 2015 | 357 | 240 | 117 | 509 | 302 | 207 | 289 | 214 | 75 |
| 2016 | 345 | 201 | 144 | 361 | 205 | 156 | 373 | 274 | 99 |
| 2017 | 215 | 168 | 47 | 233 | 158 | 75 | 318 | 239 | 79 |
| 2018 | 156 | 126 | 30 | 230 | 142 | 88 | 334 | 247 | 87 |
| 2019 | 220 | 172 | 48 | 210 | 144 | 66 | 300 | 233 | 67 |
| 2020 | 298 | 227 | 71 | 217 | 150 | 67 | 222 | 139 | 83 |
| 2021 | 404 | 318 | 86 | 248 | 170 | 78 | 391 | 297 | 94 |
| 2022 | 191 | 152 | 39 | 244 | 176 | 68 | 350 | 245 | 105 |
| 2023 | 304 | 237 | 67 | 299 | 218 | 81 | 435 | 324 | 111 |
|  |  |  |  |  |  |  |  |  |  |
| year | Schistosomiasis | Schistosomiasis | Schistosomiasis | Encephalitis | Encephalitis | Encephalitis | Rabies | Rabies | Rabies |
|  | Both gender (persons) | Male (persons) | Female (persons) | Both gender (persons) | Male (persons) | Female (persons) | Both gender (persons) | Male (persons) | Female (persons) |
| 2010 | 4522 | 2822 | 1700 | 2552 | 1524 | 1028 | 2052 | 1451 | 601 |
| 2011 | 4680 | 2789 | 1891 | 1650 | 955 | 695 | 1923 | 1349 | 574 |
| 2012 | 5006 | 2996 | 2010 | 1789 | 1072 | 717 | 1422 | 1028 | 394 |
| 2013 | 5808 | 3420 | 2388 | 2225 | 1217 | 1008 | 1180 | 818 | 362 |
| 2014 | 4516 | 2662 | 1854 | 855 | 503 | 352 | 928 | 651 | 277 |
| 2015 | 43995 | 27024 | 16971 | 628 | 352 | 276 | 801 | 587 | 214 |
| 2016 | 7616 | 5686 | 1930 | 1251 | 658 | 593 | 648 | 442 | 206 |
| 2017 | 6742 | 5068 | 1674 | 1153 | 641 | 512 | 517 | 366 | 151 |
| 2018 | 220 | 156 | 64 | 1805 | 939 | 866 | 423 | 310 | 113 |
| 2019 | 195 | 125 | 70 | 427 | 266 | 161 | 294 | 215 | 79 |
| 2020 | 52 | 36 | 16 | 291 | 185 | 106 | 200 | 132 | 68 |
| 2021 | 15 | 10 | 5 | 208 | 124 | 84 | 158 | 115 | 43 |
| 2022 | 93 | 48 | 45 | 146 | 76 | 70 | 134 | 91 | 43 |
| 2023 | 22 | 10 | 12 | 205 | 118 | 87 | 118 | 76 | 42 |

Notes: In the National Notifiable Infectious Disease Reporting System (NNIDRS) of China, "Encephalitis" typically refers to Japanese encephalitis (JE), also known as epidemic Japanese encephalitis, which is an acute zoonotic infectious disease caused by the Japanese encephalitis virus (JEV). The disease is primarily transmitted by mosquitoes and is classified as a Category B notifiable infectious disease under Chinese law, requiring mandatory reporting and surveillance.


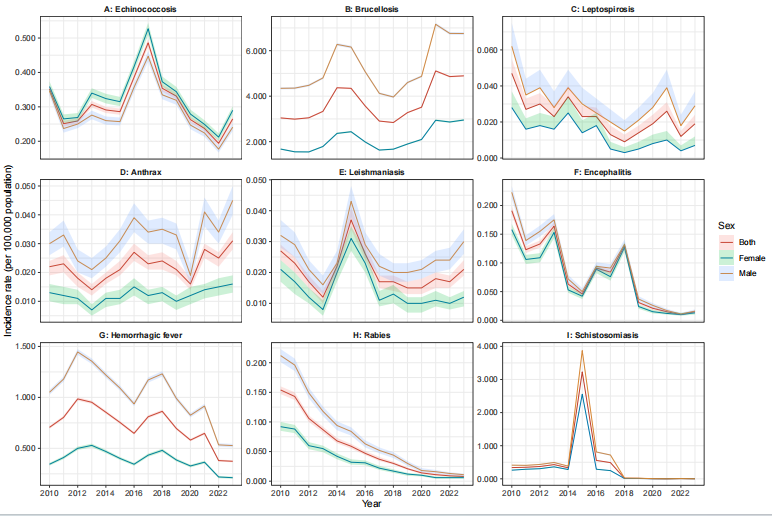


**Fig. S2**: Age–sex patterns and temporal trends of nine zoonotic diseases in the Chinese Mainland, 2010–2023(A: Echinococcosis. B: Brucellosis.C: Leptospirosis. D: anthrax. E: Leishmaniasis. F: Encephalitis.G: Hemorrhagic fever. H: Rabies. I: Schistosomiasis). In the National Notifiable Infectious Disease Reporting System of China, "Encephalitis" typically refers to Japanese encephalitis, also known as epidemic Japanese encephalitis, it is an acute zoonotic infectious disease caused by the Japanese encephalitis virus.

**Table S3**: Gender-specific distribution characteristics of nine zoonotic infectious diseases in Chinese mainland, 2010–2023.

| Index | Echinococcosis | Brucellosis | Hemorrhagic  fever | Leptospirosis | Rabies | Leishmaniasis | Anthrax | Schistosomiasis | Encephalitis |
| --- | --- | --- | --- | --- | --- | --- | --- | --- | --- |
| Age | Median (95% *CI*) | Median (95% *CI*) | Median (95% *CI*) | Median (95% *CI*) | Median (95% *CI*) | Median (95% *CI*) | Median (95% *CI*) | Median (95% *CI*) | Median (95% *CI*) |
| Both gender | 44(44, 45) | 48(38, 57) | 48(48, 49) | 51(51, 52) | 51(51, 52) | 11(9, 15) | 41(41, 42) | 50(50, 51) | 9(9, 10) |
| Female | 44(44, 45) | 59(50, 58) | 51(51, 52) | 50(49, 52) | 53(53, 54) | 3(3, 5) | 41(41, 43) | 50(50, 51) | 10(10, 11) |
| Male | 43(43, 44) | 48(37, 57) | 46(46, 47) | 52(52, 53) | 50(50, 51) | 26(24, 30) | 42(42, 43) | 49(49, 49) | 8(8, 9) |
| Z | -4.011 | 13.424 | -42.77 | -2.573 | -4.698 | -10.948 | -0.083 | -4.861 | -11.202 |
| P | <0.001 | <0.001 | <0.001 | 0.011 | <0.001 | <0.001 | 0.934 | <0.001 | <0.001 |

Notes: In the National Notifiable Infectious Disease Reporting System (NNIDRS) of China, "Encephalitis" typically refers to Japanese encephalitis (JE), also known as epidemic Japanese encephalitis, which is an acute zoonotic infectious disease caused by the Japanese encephalitis virus (JEV). The disease is primarily transmitted by mosquitoes and is classified as a Category B notifiable infectious disease under Chinese law, requiring mandatory reporting and surveillance. CI= Confidence interval.

**Table S4**: Age-specific distribution characteristics of nine zoonotic infectious diseases in Chinese mainland, 2010–2023.

| Age group | Echinococcosis n(%) | Brucellosis n(%) | Hemorrhagic  fever n(%) | Leptospirosis n(%) | Rabies n(%) | Leishmaniasis n(%) | Anthrax n(%) | Schistosomiasis n(%) | Encephalitis n(%) |
| --- | --- | --- | --- | --- | --- | --- | --- | --- | --- |
| Children  [0-5) | 355(0.606) | 5325(0.74) | 385(0.279) | 9(0.185) | 505(4.677) | 1715(44.031) | 43(1.002) | 21(0.025) | 4163(27.415) |
| Teenagers  [5-14 years) | 3333(5.69) | 10877(1.51) | 2505(1.817) | 82(1.686) | 1061(9.826) | 275(7.06) | 100(2.331) | 476(0.57) | 5020(33.059) |
| Adult [14-65 years） | 46950(80.155) | 630482(87.74) | 116468(84.476) | 3852(79.194) | 6904(63.938) | 1647(42.285) | 3856(89.883) | 74508(89.25) | 4653(30.642) |
| Elderly [65 over） | 7958(13.586) | 71873(10) | 18513(13.428) | 921(18.935) | 2328(21.56) | 258(6.624) | 291(6.783) | 8477(10.154) | 1349(8.884) |

Notes: In the National Notifiable Infectious Disease Reporting System (NNIDRS) of China, "Encephalitis" typically refers to Japanese encephalitis (JE), also known as epidemic Japanese encephalitis, which is an acute zoonotic infectious disease caused by the Japanese encephalitis virus (JEV). The disease is primarily transmitted by mosquitoes and is classified as a Category B notifiable infectious disease under Chinese law, requiring mandatory reporting and surveillance.

**Table S5**: Clinically diagnosed and laboratory-confirmed cases of nine zoonotic infectious diseases in Chinese mainland, 2010–2023.

| Index | Echinococcosis n(%) | Brucellosis n(%) | Hemorrhagic  fever n(%) | Leptospirosis n(%) | Rabies n(%) | Leishmaniasis n(%) | Anthrax n(%) | Schistosomiasis n(%) | Encephalitis n(%) |
| --- | --- | --- | --- | --- | --- | --- | --- | --- | --- |
| Clinically  diagnosed cases | 42593(72.689) | 56252(7.83) | 31212(22.639) | 2213(45.498) | 10294(95.332) | 2934(75.327) | 3583(83.52) | 69844(83.664) | 2081)13.704 |
| Laboratory confirmed cases | 16003(27.311) | 662307(92.17) | 106659(77.361) | 2651(54.502) | 504(4.668) | 961(24.673) | 707(16.48) | 13638(16.336) | 13104)86.296 |

Notes: In the National Notifiable Infectious Disease Reporting System (NNIDRS) of China, "Encephalitis" typically refers to Japanese encephalitis (JE), also known as epidemic Japanese encephalitis, which is an acute zoonotic infectious disease caused by the Japanese encephalitis virus (JEV). The disease is primarily transmitted by mosquitoes and is classified as a Category B notifiable infectious disease under Chinese law, requiring mandatory reporting and surveillance.

**Table S6**: Modeling the incidence rates of nine common zoonotic diseases in Chinese mainland, 2024–2035.

| Disease | Model | R-squared | RMSE | MAPE | Normalized BIC | Variable | Estimate | SE | *t* | *P* |
| --- | --- | --- | --- | --- | --- | --- | --- | --- | --- | --- |
| Echinococcosis | ARIMA(1,0,0) | 0.286 | 0.065 | 16.049 | -5.098 | AR | 0.535 | 0.242 | 2.213 | 0.047 |
| Echinococcosis | Holt's linear trend | 0.112 | 0.072 | 16.962 | -4.88 | Alpha (Level) | 0.999 | 0.315 | 3.171 | 0.008 |
| Brucellosis | ARIMA(1,0,0) | 0.441 | 0.642 | 11.859 | -5.508 | AR | 0.707 | 0.228 | 3.103 | 0.009 |
| Hemorrhagic fever | ARIMA(1,0,0) | 0.526 | 0.133 | 16.584 | -3.664 | AR | 0.794 | 0.238 | 3.341 | 0.006 |
| Hemorrhagic fever | Holt's linear trend | 0.522 | 0.133 | 15.523 | -3.655 | Alpha (Level) | 0.999 | 0.282 | 3.537 | 0.004 |
| Hemorrhagic fever | Holt's linear trend |  |  |  |  | Gamma (Trend) | 1.829E-5 | 0.099 | 0 | 0.998 |
| Leptospirosis | ARIMA(1,0,0) | 0.190 | 0.01 | 33.622 | -8.764 | AR | 0.525 | 0.241 | 2.177 | 0.05 |
| Leptospirosis | Brown's linear trend | 0.113 | 0.01 | 33.52 | -9.047 | Alpha (Level and Trend) | 0.321 | 0.111 | 2.881 | 0.013 |
| Leishmaniasis | Damped trend | 0.109 | 0.005 | 17.527 | -10.439 | Alpha (Level) | 0.09 | 0.286 | 0.315 | 0.759 |
| Leishmaniasis | Damped trend | 0.104 | 1.289 | 686.307 | 1.073 | Gamma (Trend) | 1.361E-5 | 1.045 | 1.303E-5 | 1 |
| Leishmaniasis | Damped trend |  |  |  |  | Phi (Trend damping factor) | 1 | 0.287 | 3.479 | 0.005 |
| Anthrax | Brown's linear trend | 0.109 | 0.005 | 17.527 | -10.439 | Alpha (Level and Trend) | 0.223 | 0.087 | 2.546 | 0.024 |
| Schistosomiasis | Damped Trend | 0.109 | 0.005 | 17.527 | -10.439 | Alpha (Level) | 0.782 | 0.315 | 2.48 | 0.031 |
| Schistosomiasis | Damped Trend | 0.104 | 1.289 | 686.307 | 1.073 | Gamma (Trend) | 0 | 0.266 | 0.001 | 0.999 |
| Schistosomiasis | Damped Trend |  |  |  |  | Phi (Trend damping factor) | 0.993 | 0.07 | 14.274 | <0.001 |
| Encephalitis | ARIMA(1,0,0) | 0.274 | 0.054 | 96.652 | -5.475 | AR | 0.687 | 0.221 | 3.115 | 0.009 |
| Rabies | Holt's linear trend | 0.975 | 0.008 | 16.315 | -9.258 | Alpha (Level) | 0.597 | 0.202 | 2.956 | 0.012 |
|  |  |  |  |  |  | Gamma (Trend) | 1 | 0.596 | 1.677 | 0.119 |

Notes: In the National Notifiable Infectious Disease Reporting System (NNIDRS) of China, "Encephalitis" typically refers to Japanese encephalitis (JE), also known as epidemic Japanese encephalitis, which is an acute zoonotic infectious disease caused by the Japanese encephalitis virus (JEV). The disease is primarily transmitted by mosquitoes and is classified as a Category B notifiable infectious disease under Chinese law, requiring mandatory reporting and surveillance. AR=Autoregressive. ARIMA=Autoregressive integrated moving average. MAPE=Mean absolute percentage error. RMSE= Root mean square error. NBIC=Normalized Bayesian Information Criterion. SE=Standard Error.

**Table S7**: Projected values and temporal trends of zoonotic diseases in Chinese mainland, 2024–2035.

| Disease | Model | Variable | Relative error (mean)  % 2010－2023 | Relative error (average)  %  2010－2023 | 2035 year  Incidence rate (per 100,000 population, 95% *CI)* | EAPC (%) 2023－2035 | AAPC (%) 2024－2035 |
| --- | --- | --- | --- | --- | --- | --- | --- |
| Echinococcosis | ARIMA(1,0,0) | AR | 0.468 | 0.462 | 0.306(0.178, 0.498) | 0.44(0.16,0.72) | 0.440(0.127, 0.753) |
| Echinococcosis | Holt's linear trend | Alpha (Level) | 16.962 | 18.361 | 0.218(0.0.762) | -1.62(-1.650,-1.591) | -1.619(-1.624, -1.615) |
| Brucellosis | ARIMA(1,0,0) | AR | 11.856 | 8.163 | 3.811(2.267, 6.087) | -1.435(-1.914,-0.953) | -0.068(-0.073,-0.063 |
| Hemorrhagic fever | ARIMA(1,0,0) | AR | 16.584 | 13.554 | 0.640(0.284, 1.277) | 3.503(2.590,4.424) | 3.8646(3.6625, 4.0922) |
| Hemorrhagic fever | Holt's linear trend | Alpha (Level) | 24.340 | 20.955 | 0.197(0.001, 0.848) | -5.243(-5.286,-5.200) | -5.2212(-5.2353, -5.2096) |
| Hemorrhagic fever | Holt's linear trend | Gamma (Trend) |  |  |  |  |  |
| Leptospirosis | ARIMA(1,0,0) | AR | 33.622 | 20.945 | 0.027(0.009, 0.064) | 0.582(0.202,0.963) | 0.8695(0.7644, 0.9848) |
| Leptospirosis | Brown's linear trend | Alpha (Level and Trend) | 33.520 | 34.349 | 0.013(0.001, 0.101) | -3.394(-3.518,-3.269) | -3.4068(-3.4250, -3.3885) |
| Leishmaniasis | Damped trend | Alpha (Level) | 24.340 | 20.955 | 0.0122(0.001, 0.029) | -3.020(-3.118,-2.921) | -3.0198(-3.1452, -2.8944) |
| Leishmaniasis | Damped trend | Gamma (Trend) |  |  |  |  |  |
| Leishmaniasis | Damped trend | Phi (Trend damping factor) |  |  |  |  |  |
| Anthrax | Brown's linear trend | Alpha (Level and Trend) | 17.527 | 13.324 | 0.035(0.008, 0.062) |  | 2.178(2.127,2.229 |
| Schistosomiasis | Damped Trend | Alpha (Level) | 3256.309 | 3163.819 | 0.09(0.000, 0.142) | 34.01(33.58,34.44) | 33.9884(33.8987, 34.0859) |
| Schistosomiasis | Damped Trend | Gamma (Trend) |  |  |  |  |  |
| Schistosomiasis | Damped Trend | Phi (Trend damping factor) |  |  |  |  |  |
| Encephalitis | ARIMA(1,0,0) | AR | 96.652 | 53.654 | 0.084(0.000, 0.232) | 0.582(0.202,0.963) | 0.8695(0.7644, 0.9848) |
|  |  | Gamma (Trend) |  |  |  |  |  |

Notes: In the National Notifiable Infectious Disease Reporting System (NNIDRS) of China, "Encephalitis" typically refers to Japanese encephalitis (JE), also known as epidemic Japanese encephalitis, which is an acute zoonotic infectious disease caused by the Japanese encephalitis virus (JEV). The disease is primarily transmitted by mosquitoes and is classified as a Category B notifiable infectious disease under Chinese law, requiring mandatory reporting and surveillance. AAPC=Average annual percent change. CI=Confidence interval. EAPC= Estimation of the annual percentage change.
